# Fermentation with Lactic Acid Bacteria for Bean Flour Improvement: Experimental Study and Molecular Modeling as Complementary Tools

**DOI:** 10.3390/foods13132105

**Published:** 2024-07-02

**Authors:** Carlos Sabater, Gabriel D. Sáez, Nadia Suárez, Marisa S. Garro, Abelardo Margolles, Gabriela Zárate

**Affiliations:** 1 Department of Microbiology and Biochemistry of Dairy Products, Dairy Research Institute of Asturias (IPLA), Spanish National Research Council (CSIC), Paseo Río Linares S/N, 33300 Villaviciosa, Asturias, Spain; carlos.sabater@csic.es (C.S.); amargolles@ipla.csic.es (A.M.); 2 Health Research Institute of Asturias (ISPA), 33011 Oviedo, Asturias, Spain; 3 Laboratory of Technological Ecophysiology, Reference Centre for Lactobacilli (CERELA-CONICET), Chacabuco 145, San Miguel de Tucumán 4000, Tucumán, Argentina; gabrieldsaez@yahoo.com.ar (G.D.S.); nsuarez@cerela.org.ar (N.S.); mgarro@cerela.org.ar (M.S.G.); 4 Department of Food Microbiology, University of San Pablo Tucumán, Av. Solano Vera y Camino a Villa Nougués, San Pablo 4129, Tucumán, Argentina

**Keywords:** beans, fermentation, lactic acid bacteria, comparative genomics, molecular modeling

## Abstract

Pulses are considered superfoods for the future world due to their properties, but they require processing to reduce antinutritional factors (ANFs) and increase bioactivity. In this study, bean flour (*Phaseolus vulgaris* L.) was fermented under different conditions (addition of *Lactiplantibacillus plantarum* CRL 2211 and/or *Weissella paramesenteroides* CRL 2182, temperature, time and dough yield) to improve its nutri-functional quality. Fermentation for 24 h at 37 °C with the mixed starter increased the lactic acid bacteria (LAB) population, acidity, polyphenol content (TPC) and ANF removal more than spontaneous fermentation. Statistical and rep-PCR analysis showed that fermentation was mainly conducted by *Lp. plantarum* CRL 2211. Metabolic modeling revealed potential cross-feeding between *Lp. plantarum* and *W. paramesenteroides*, while the molecular docking and dynamic simulation of LAB tannases and proteinases involved in ANF removal revealed their chemical affinity to gallocatechin and trypsin inhibitors. Fermentation was better than soaking, germination and cooking for enhancing bean flour properties: it increased the free amino acids content by 50% by releasing glutamine, glutamic acid, arginine, leucine and lysine and modified TPC by increasing gallic acid and decreasing caffeic, ferulic and vanillic acids and quercetin-3-glucoside. The combination of experimental and simulation data may help us to understand fermentation processes and to design products with desirable features.

## 1. Introduction

Beans (*Phaseolus vulgaris* L. varieties) are economically relevant food crops worldwide. In addition to providing nutrients such as carbohydrates, proteins, dietary fiber, minerals and vitamins, beans also contain diverse phytochemicals such as polyphenols and peptides, which confer numerous physiological and health benefits. As a result, beans may be considered superfoods. The consumption of beans has been associated with the prevention of cardiovascular disease, obesity, metabolic syndrome, diabetes, cancer and other non-communicable diseases [1]. Furthermore, pulse-derived products have been considered as a high-nutritional gluten-free alternative for individuals with celiac disease [2]. Their significance in global nutrition has led the United Nations to recognize legumes as a source of nutritious seeds for a sustainable future and to designate 2016 as the International Year of Pulses [3].

Pulses are generally regarded as a significant source of high-quality protein in developing countries where a considerable proportion of the population has limited access to meat protein. Currently, a variety of beans (*Phaseolus vulgaris* L., *P. lunatus*, *P. coccineus* and *Vigna savi*) are consumed as staple foods in American countries, with an intake that varies considerably between 1 and 20 kg/person/year [4]. Beans are commonly consumed as unprocessed seeds, canned products and gluten-free flour derivatives [2]. Nevertheless, these raw materials require some technological processing to enhance their nutritional value due to the presence of antinutritional factors (ANFs), including protease inhibitors, tannins, lectins and phytic acid among others, which reduce nutrient absorption, palatability and induce intestinal discomfort [5]. Different food processing techniques are employed to inactivate ANFs including soaking, dehulling, germination, heating and high pressure. However, the bioactivity of phytochemicals may be negatively affected by these treatments, and residual ANFs may remain after processing [6]. On the contrary, fermentation with lactic acid bacteria (LAB) is a suitable alternative for enhancing the nutri-functional quality of pulses [5]. A number of studies have demonstrated that the enzyme activities of LAB are involved in the removal of ANF, thereby protecting the consumer from their deleterious effects [7,8]. Furthermore, fermentation can enhance the organoleptic properties (flavor and texture) and safety (through antimicrobial production) of pulses and may facilitate the release of bioactive metabolites, thereby conferring health benefits [9,10,11,12]. The fermentation process may be carried out spontaneously or may be controlled by the inoculation of selected starters. In this context, LAB obtained from the food matrix of interest are generally the microorganisms of choice because they are generally recognized as safe (GRAS status) and already well adapted to these raw materials [13].

In recent years, many computational approaches have emerged to complement experimental data and gain insight into molecular interactions that could explain complex physicochemical and biological phenomena. Molecular modeling has attracted attention in the field of food science, with the aim of predicting the behavior of enzymes, cryoprotectants and carbohydrates, among other molecules. It has also been employed to reconstruct in silico the metabolic capabilities of food/gut microbiota [14,15].

In previous studies, we isolated and characterized LAB from legumes and other vegetables consumed in Argentina and selected the most suitable strains for obtaining novel fermented products [16,17,18]. Two strains identified as *Lactiplantibacillus plantarum* CRL 2211 and *Weissella paramesenteroides* CRL 2182 were selected for their techno-functional and safety properties to enhance the quality of legume flours. In this study, the effect of various parameters relevant in sourdough technology, including dough yield, temperature, time and the inoculation of starters, on the nutri-functional quality of fermented bean doughs was evaluated. Molecular dynamic simulations and metabolic modeling were employed as complementary techniques to elucidate the enzymatic behavior and microbial interactions occurring during the fermentation process. Furthermore, fermentation was compared with other processing methods to identify the optimal strategy for enhancing the quality of this food matrix.

## 2. Materials and Methods

### 2.1. Microorganisms and Culture Conditions

*Lp. plantarum* CRL 2211 and *W. paramesenteroides* CRL 2182 (identified by 16S rDNA, ENA database, accession numbers LT714203 and LT900386) were provided by the culture collection of CERELA-CONICET. These strains were previously characterized and selected by their ability to grow on legume extracts (Figure S1, Supplementary Materials), acidifying, proteolytic, tannase (EC 3.1.1.20) and gallate decarboxylase (EC 4.1.1.59) activities and pathogen inhibition that make them suitable for the fermentation of legume-based substrates [16,17,18,19]. Cultures grown in De Man, Rogosa, Sharpe broth (MRS, Britania, Buenos Aires, Argentina) (16 h, 37 °C) were washed (7000× *g*, 15 min) and resuspended in NaCl 0.85% at a concentration of 10^8^ CFU/mL.

### 2.2. Experimental Design for Beans Flour Fermentation

The addition of the starter cultures *Lp. plantarum* CRL 2211 (A) and/or *W. paremesenteroides* CRL 2182 (B), temperature (C), time of fermentation (D) and the dough yield (E) were the fermentation variables selected to evaluate their incidence on bean flour quality. For this purpose, a Randomized Complete Block Design (RCBD): 2^5^ was configured (Table S1). Two levels were assigned to each variable (A, B: 0 and 7 log CFU/g; C: 30 and 37 °C; D: 8 and 24 h; E: 160 and 200) so that the experimental model was composed of 32 runs divided into 4 blocks. No repetitions were made under the assumption that higher-order interactions are not statistically significant and are included in the experimental error. The measured responses, microbial development (Y_1_), acidification (Y_2_), phenolic compounds (Y_3_), tannins (Y_4_), and trypsin inhibitor contents (Y_5_), were defined as follows: Y_1_ was the difference between the final and initial microbial counts for each fermentation condition; Y_2_ was the pH difference between fermented and uninoculated doughs (control) and Y_3_, Y_4_ and Y_5_ were the differences in the concentrations of the analyzed compounds from fermented doughs at the final time of fermentation and the control.

### 2.3. Raw Material and Dough Formulation

Alubia bean flours were obtained through the mechanical milling of grains provided by INTA-Salta (National Institute of Agricultural Technology, Salta, Argentina). Flours (proximate composition (%): moisture, 9.4; protein 32.3; fat, 1.04; ash, 5.6; fiber, 1.7; carbohydrates, 51.0, Table S2) were mixed with tap water to obtain the different dough yields [(dough mass/flour mass) × 100] tested. The mixtures were homogenized, distributed into sterile flasks (100 g of wet weight dough), inoculated (10^7^ CFU/g) and incubated according to the experimental conditions of each run. Samples for response analysis (Y_1_ to Y_5_) were taken as stated in the RCBD model at different times (0, 8 and 24 h).

### 2.4. Microbiological Analysis and pH Determinations

Ten grams of dough samples was placed in sterile bags with 90 mL of 0.85% NaCl, homogenized in a Stomacher lab-blender 400 (Seward Medical, London, UK), ten-fold diluted and spread on MRS agar supplemented with 0.1 g/L ciclohexymide (Sigma, St. Louis, MO, USA) and incubated for 48 h at 37 °C under microaerophilic conditions. After counting, all the colonies (approx. 20 CFU) from the highest dilution (10^−7^) were picked from plates and subjected to genotypic characterization by rep-PCR (Repetitive element palindromic-Polymerase Chain Reaction) analysis using the (GTG)_5_ (5′-GTGGTGGTGGTGGTG-3′) primer and the conditions reported by Sáez et al. [16]. Rep-PCR fingerprints were analyzed using GelJ Software, v2.0 [20]. The acidifications of doughs were followed by pH measurements performed with a pH-meter probe (Altronix TPX I, New York, NY, USA).

### 2.5. Phenolics’ and Tannins’ Quantification

Total phenolic compounds (TPCs) and tannins’ content were determined by spectrophotometry as described by Sáez et al. [16]. Polyphenols were extracted from doughs with 70% methanol and ultrasonic treatment (300 W) for 20 min at room temperature. Sample supernatants reacted with 1 N Folin Ciocalteau reagent (Anedra, Buenos Aires, Argentina), and absorbances at 725 nm (Versamax Spectrophotometer, Molecular Devices, San José, CA, USA) were determined before (total polyphenols) and after treatment with polyvinylpolypyrrolidone (non-tannin phenolic compounds) (PVPP; Sigma, St. Louis, MO, USA). Tannin contents were calculated as the difference between total phenolics and non-tannin phenolics and expressed as mg GAE/100 g according to a calibration curve constructed with gallic acid as the standard. The polyphenol profile was determined in ethanolic extracts of raw, spontaneously and controlled fermented doughs by mass spectrometry coupled with liquid chromatography (LC-MS). Mass spectra were obtained using a Shimadzu Nexera X2 HPLC system coupled with a Sciex QTRAP 6500+ mass spectrometer (Sciex, Framingham, MA, USA). The MS/MS system was operated with the IonDriveTM Turbo V ion source (ESI) in positive and negative ion modes (Sciex, Framingham, MA, USA) in the conditions described by Vu and Alvarez [21]. The compounds were identified by the interpretation of their UV and mass spectra as well as by comparison with reference standard compounds (apigenin, caffeic acid, chlorogenic acid, cinnamic acid, daidzein, ferulic acid, gallic acid, genistein, hesperetin, kaempferol, luteolin, naringenin, p-coumaric acid, phloretin, protocatechuic acid, quercetin, quercetin-3-galactoside, quercetin-3-glucoside, resveratrol, rutin, syringic acid, vanillic acid from Sigma, St. Louis, MO, USA, Table S3).

### 2.6. Enzyme Inhibitors’ Quantification

The presence of enzyme inhibitors in the doughs extracts was determined by spectrophotometric methods [19]. Aliquots of extracts were added to reactions containing the enzymes trypsin (type III, from bovine pancreas 20 mg/L), α-chymotrypsin (40 µg/mL) or α-amylase (30 µg/mL) and their respective substrates, N-benzoyl-DL-arginine p-nitroanilide (BAPNA, 40 mg/mL), casein (1% *w*/*v*) and starch (1% *w*/*v*) solution. The inhibitors’ activity was expressed as units of enzyme inhibited per gram of sample (TIA mg/g; CUI/g and AUI/g, respectively).

### 2.7. Determination of Free Amino Acids (FAAs)

Total and individual FAAs were determined on methanolic extracts by high-performance liquid chromatography using Shimadzu Prominence equipment (Shimadzu Corporation, Tokyo, Japan) provided with a Gemini 5 µm C18 column (110 Å—150 × 4.6 mm, Phenomenex, Torrance, CA, USA) and commercial AA as standards. Elution was at 40 °C, with a flow rate of 1.0 mL/min, using NaH_2_PO_4_ (40 mM) pH 6.4 as mobile phase A and CH_3_CN/MeOH/H_2_O (45:45:10) as phase B. Fluorescence detection wavelengths were Ext: 340 nm–Em: 450 nm.

### 2.8. In Silico Analyses

#### 2.8.1. Genome Sequencing of *Lp. plantarum* CRL 2211 and *W. paramesenteroides* CRL 2182

The genome sequencing of the *Lp. plantarum* CRL 2211 and *W. paramesenteroides* CRL 2182 strains was performed at GenProbio S.R.L. (Parma, Italy), with an Illumina MiSeq Sequencing System. The quality control of paired-end sequencing reads was performed using fastq-mcf (v.1.04.807). Then, pre-processed reads were assembled using SPAdes (v.3.15.5), and genome contig reordering based on their sequence length was performed using bwa (v.0.7.17-r1188) and samtools (v. 1.6.0). The genome assemblies of the *Lp. plantarum* CRL 2211 and *W. paramesenteroides* CRL 2182 strains were annotated following the pipelines described in Section 2.8.2. The raw sequences’ data were deposited in the Sequence Read Archive (SRA) of the NCBI (https://www.ncbi.nlm.nih.gov/sra, accessed on 24 May 2024) under Bio Project code PRJNA911216 and BioSample accessions SAMN32166586 and SAMN32166587.

#### 2.8.2. Metabolic Modeling

A comparative genomics study of LAB species that may be present in fermented bean flour according to the literature [16,22] was performed in silico. For this purpose, reference genomes and genomic assemblies from *Companilactobacillus alimentarius* (n = 2), *Fructilactobacillus sanfranciescensis* (n = 21), *Levilactobacillus brevis* (n = 54), *Lentilactobacillus hilgardii* (n = 6), *Lactococcus lactis* (n = 164), *Lp. plantarum* (n = 416), *Limosilactobacillus reuteri* (n = 176) and *W. paramesenteroides* (n = 25) were retrieved from the ENA repository (https://www.ebi.ac.uk/ena/browser/search, accessed on 24 May 2024). In addition, the genome assemblies of *Lp. plantarum* CRL 2211 and *W. paramesenteroides* CRL 2182 were included in this study (see Section 2.8.1). Sequences were annotated using HMMER software v.3.3 and the Pfam database [23] to identify functional domains associated with carbohydrate and lipid metabolism, proteolysis and tannin hydrolysis involved in sourdough fermentation [24,25,26]. Then, potential cross-feeding interaction mechanisms between these LAB species were elucidated using metage2metabo v.1.5.0 software [27]. With this aim, a metabolic model of alubia beans (*Phaseolus vulgaris* L.) containing all metabolites present in this substrate was retrieved from the ModelSEED web portal (https://modelseed.org/, accessed on 24 May 2024) and used as seed for cross-feeding computations.

#### 2.8.3. Molecular Modeling

The molecular modeling of enzymatic reactions involved in tannin hydrolysis and trypsin inhibitor removal during alubia bean fermentation was carried out. For this purpose, microbial serine-type endopeptidases and tannases from LAB reported in legumes such as *Lp. plantarum*, *L. lactis* and *Lv. brevis* [16,22], as well as Bowman–Birk-type proteinase inhibitors, were collected from the official UNIPROT repository (https://www.uniprot.org/, accessed on 24 May 2024) (Table S4). Gallocatechin structure, a naturally occurring phenolic compound in beans [28], was retrieved from the ChemSpider database (http://www.chemspider.com/, accessed on 24 May 2024). Then, the molecular docking of tannase–phenol complexes was performed in Autodock Vina v.1.2.5 software [29], while the protein–protein docking of peptidase–inhibitor complexes was performed on the HawkDock server (http://cadd.zju.edu.cn/hawkdock/, accessed on 24 May 2024) [30]. Homology modeling [31] and active site prediction [32] were employed when 3D structures were not available in the official databases.

To calculate accurate enzyme–substrate binding energies, molecular dynamic simulations of docked complexes were performed. Binding energies for endopeptidase–trypsin inhibitor complexes were determined by MM/GBSA method [29], while the MM/PBSA method [33] implemented in GROMACS v.2024.1 software [34] was used for tannase–polyphenol complexes. Input files were generated using CHARMM-GUI v.3.8 (http://www.charmm-gui.org/, accessed on 24 May 2024). Simulations were equilibrated for 125 ps and run for 10 ns at 30 °C in explicit solvent.

### 2.9. Other Processing Treatments

Soaking, germination and cooking (by boiling and microwave) were applied to beans before grinding them to flour as described in Sáez et al. [19]. Grains were soaked in distilled water (1:10, *w*/*v*, 12 h, 25 °C) and then drained. For germination, beans were kept between thick layers of cotton cloth in the dark for 3 days at 25 °C. In the case of cooking, the rinsed seeds were boiled (100 °C) in tap water for 90 min or cooked in a microwave oven (MPR8520 Model, Philco, Buenos Aires, Argentina) on high (700 W, 120 °C), for 15 min. Raw and treated legumes were dried overnight at 50 °C, ground to pass through a 60-mesh sieve and stored in screw cap plastic containers at 4 °C. Phenolics, enzyme inhibitors and FAAs were determined as described in Section 2.5, Section 2.6 and Section 2.7.

### 2.10. Antioxidant Activity

The antioxidant capacity of bean dough extracts was determined by the DPPH (Sigma, St. Louis, MO, USA) radical scavenging assay [19]: 0.1 mL of extract was mixed with 3.9 mL of methanolic DPPH solution (6 × 10^−5^ mol/L), vigorously shaken and allowed to stand at room temperature in the dark for 20 min. The decrease in the absorbance of the resulting solution was determined at 515 nm (UV–visible Spectrophotometer, Varioskan Flash; Thermo Fisher Scientific, Cambridge, MA, USA). The control was prepared using methanol instead of the extract, and its absorbance was measured at *t* = 0. The percentage of inhibition of the DPPH radical by the samples was calculated as follows: % DPPH scavenging = (1 − Abs sample_t20_/Abs control_t0_) × 100.

### 2.11. Statistical Analysis of Data

RCBD was performed once under the assumption that higher-order interactions are included in the experimental error. Fermentation under defined conditions and other processing treatments were performed three times and the results expressed as mean values ± standard deviation. Pearson correlation coefficients, ANOVA tests and Tukey’s test for *p* < 0.05 were calculated for all data generated (Minitab 17 statistical software, MINITAB Inc., State College, PA, USA).

## 3. Results and Discussion

LAB-fermented legumes could be attractive novel functional ingredients or foods, with improved organoleptic properties and less ANFs, but the correct selection of microorganisms as starters is a crucial step to take into account. In a previous study [19], we demonstrated that *Lactiplantibacillus plantarum* CRL 2211 and *Weisella paramesenteroides* CRL 2182 were appropriate to improve by fermentation the nutritional and technological properties of chickpea flours which allow for us to obtain derived cookies with higher antioxidant activity than their unfermented counterparts. Since their potential in other legume flours is unknown, the fermentation of locally produced alubia bean flours with these legume strains was carried out.

**Figure 1:**
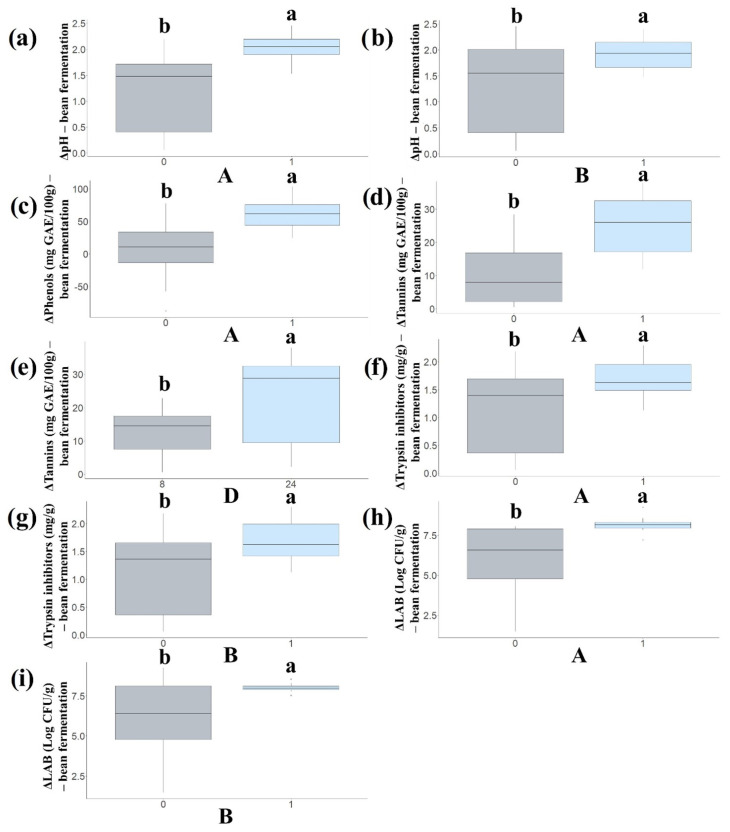
Boxplots showing the influence of independent variables (A: *Lactiplantibacillus plantarum* CRL 2211, B: *Weissella paramesenteroides* CRL 2182, D: time, h) on alubia bean flour ΔpH (**a**,**b**), Δtotal phenolic content (mg GAE/100 g); (**c**), Δ tannins (mg GAE/100 g); (**d**,**e**) and Δ trypsin inhibitor contents (mg/g); (**f**,**g**) and Δ LAB counts (Log CFU/g); (**h**,**i**). ^a,b^ Statistically significant differences (*p* < 0.05).

A factorial experimental model of RCBD was used to evaluate the effect of some fermentation conditions on relevant quality parameters. Among independent variables, the addition of *Lp. plantarum* CRL 2211 (A), *W. paramesenteroides* CRL 2182 (B) and the treatment time (D) were positively correlated to all the response variables (Figure S2). Fermentation time was highly correlated to tannin hydrolysis, whereas no significant correlation was found for temperature or dough yield with any flour quality indicators (Figure S2). In agreement, Figure 1 shows that these variables (A, B and D) led to statistically significant (*p* < 0.05) responses between the two levels studied.

### 3.1. Effect of Process Variables on Dough Acidification

Dough acidification is relevant because it modulates enzymes involved in flavor and bioactive production, impacts the organoleptic properties and controls the undesirable microbiota [12,25]. The inoculation of doughs with each strain and mixed starter cultures resulted in a significantly higher (*p* < 0.05) acidification of bean flour (Figure 1a,b) since the pH decreased from 6.40 to 3.75 in mixed LAB-fermented doughs but reached a final pH of 4.93 in the spontaneously fermented flours (Table 1, runs 8, 32). Pareto charts (Figure S3a) show that three factors, *Lp. plantarum* CRL 2211 (A), *W. paramesenteroides* CRL 2182 (B) and the interaction of AxB, have *p*-values lower than 0.05, indicating that they are significantly different from zero at the 95% confidence level. The deltas of pH values ranging from 1.48 to 2.45 (runs 4, 8) (in contrast to 0.06 to 1.47 units in unstarted doughs, runs 1, 32) indicate that the addition of single strains as well as both combined contribute to the higher efficiency of the acidification process (Table 1, Figure 1a,b and Figure S3b). The same mixed starter added to chickpea flour acidified the dough less (from 6.4 to 4.0) [19]. Other LAB co-cultures, such as *Lp. plantarum* C48 and *L. brevis* AM7 used as starters for legumes sourdough fermentation, also decreased pH to 4.0–4.4, significantly lower than the control doughs [35]. In the same manner, fermentation with single (*Lp. plantarum*) or mixed culture (consortium of *Lp. plantarum*, *Oenococcus oeni*, *Saccharomyces cerevisiae* and *Acetobacter aceti*) led to a strong acidification of cowpea bean flours after 4 days of fermentation (pH decreased from 6.0 to 4.65), showing that acidification will depend on the metabolic capacity of the starters and also the chemical composition of the food matrix [36].

### 3.2. Effect of Process Variables on TPC

Phenolic compounds are secondary metabolites widely distributed in plants that exert beneficial physiological effects by preventing oxidative stress [37]. In consequence, the increase in these phytochemicals in food is a desirable property. Fermentation with the selected LAB led to the higher (*p* < 0.05) TPC in the matrix (a maximum of 382.14 mg GAE/100 g for LAB-fermented flours vs. 312.50 mg GAE/100 g for spontaneously fermented flours) (Table 1 runs 2, 32, Figure 1c).

Although the increase in TPC in sourdoughs was evidenced after fermentation with both strains used as single or mixed cultures (Table 1), *Lp. plantarum* CRL 2211 displayed better performance when compared to *W. paramesenteroides* CRL 2182, as shown by variable analysis (Figure 1c and Figure S3c), whereas the rest of the experimental variables studied did not exert any relevant effect. This effect can be attributed to the presence in *Lp. plantarum* CRL 2211 of tannase (EC 3.1.1.20) and gallate decarboxylase (EC 4.1.1.59) activities that allow this microorganism to hydrolyze complex molecules of phenolic compounds, whereas *W. paramesenteroides* CRL 2182 only exhibits gallate decarboxylase activity [16]. The increase in total phenols after the fermentation of legumes with the selected *Lp. plantarum* strains has been reported for cowpea beans, chickpea, lentils and pea flour [35,38,39]. In most of the cases, the increases in total phenols were in the range of 20–70%. It has been reported that *Lp. plantarum* increases the concentration of phenolic compounds in the food matrix by the hydrolysis of complex phenolics into simpler compounds with greater activity [37].

### 3.3. Effect of Process Variables on Tannin Hydrolysis

Tannins are phenolic compounds that form complexes with dietary proteins, reducing their absorption and bioavailability in the intestinal tract, so their elimination would be desirable in legume-derived foods. As reported for TPC, fermentation with *Lp. plantarum* CRL 2211 (A) resulted in a greater (*p* < 0.05) removal of tannins, and this effect increased at prolonged fermentation times (24 h) (up to 37.93 mg GAE/100 g of tannins at 24 h vs. 0.52 mg GAE/100 g at 8 h for unstarted doughs) (Table 1 runs 15, 20 and Figure 1d,e). Figure S3d shows that the *Lp. plantarum* CRL 2211 (A), *W. paramesenteroides* CRL 2182 (B) and fermentation time (D) variables showed statistically significant *p*-values and that the A × B interaction also increased on prolonged times of treatment. Coda et al. [7] and Curiel et al. [35] reported a decrease in tannins and other ANFs of some legume flours when they were fermented with selected strains of *Lp. plantarum* and *L. brevis*. In this work, *Lp. plantarum* CRL 2211 alone or combined with *W. paramesenteroides* CRL 2182 was able to eliminate tannins almost completely, and this effect increased over time (24 h). As was reported previously, this decrease may be due to the tannase and gallate decarboxylase activities of the starters [16].

### 3.4. Effect of Process Variables on Removal of Trypsin Inhibitors

Trypsin inhibitors decrease the digestibility of proteins and mineral absorption affecting the consumer’s nutritional status, so different strategies have been applied to remove these ANFs from vegetables [6]. Fermentation with *Lp. plantarum* CRL 2211 (A) and *W. paramesenteroides* CRL 2182 (B) as single and co-cultures (Figure 1f,g and Figure S3e) led to a higher removal of trypsin inhibitors than spontaneous fermentations (*p* < 0.05), and the effect increased at prolonged fermentation times (Table 1, runs 2, 17, 21). In contrast, *W. paramesenteroides* CRL 2182 was not able to remove trypsin inhibitors from chickpea flours [19]. Other studies have reported that bacterial (*Bacillus* and some LAB) and fungal (*Aspergillus*, *Sacharomyces*) fermentations decrease trypsin inhibitors content in grains by 30 to 80%, which could be due to microbial proteolytic activities that degrade unwanted substances increasing amino acid bioavailability [7,8,19].

### 3.5. Effect of Process Variables on Dough LAB Microbiota

Finally, the significance of the different variables on the lactic bacterial growth was analyzed. The Pareto chart (Figure S3a) shows that variables A, B and the interactions of A × B and B × E had a significant effect on LAB population, according to *p*-values. In other words, the addition of *Lp. plantarum* CRL 2211 and *W. paramesenteroides* CRL 2182 as single or mixed starter cultures contributed to the high prevalence of LAB in the fermented doughs’ microbiota (7.61–9.64 Log CFU/g, Table 1, runs 25, 30). Without a starter culture, the wild LAB microbiota reached 1.88–6.00 Log CFU/g (Table 1, runs 7, 12). When *Lp. plantarum* C48 and *Lv. brevis* AM7 were used as starters for legumes’ sourdough fermentation, Curiel et al. [35] observed that the cell density of presumptive LAB reached 9.8–10.2 Log CFU/g after 24 h, whereas *Lp. plantarum* VTT E-133328 inoculated to faba bean flour led to 9.4 ± 0.5–9.6 ± 0.2 Log CFU/g of LAB at the end of fermentation.

**Figure 2:**
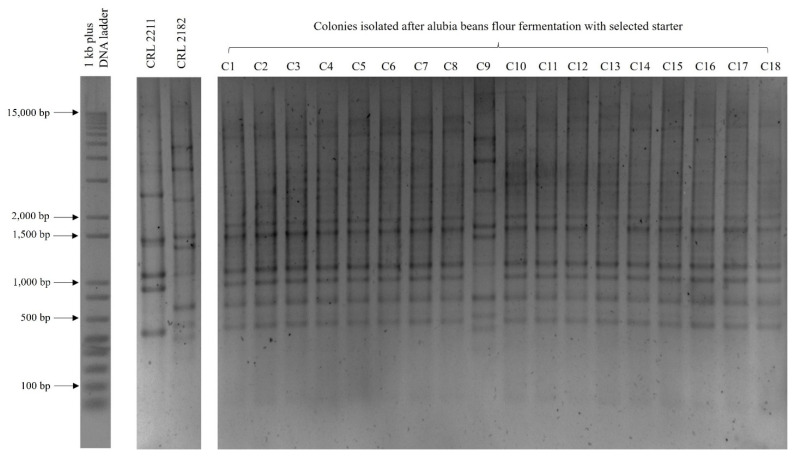
Repetitive element palindromic-PCR (rep-PCR) profiles of lactic acid bacteria (LAB) colonies isolated from alubia bean sourdough inoculated with *Lp. plantarum* CRL 2211 and *W. paramesenteroides* CRL 2182 and fermented for 24 h at 37 °C.

Based on statistical results, fermentation with both strains at longer time (24 h) was selected as relevant for increasing LAB population, acidity, TPC and removing ANFs from bean flour. The higher temperature (37 °C) and lower DY (160) were additionally chosen. For monitoring the starter cultures, rep-PCR analysis was performed with all the colonies grown on MRS agar after fermentation under the defined conditions. The comparison of the profiles obtained with that of the starter cultures showed that both microorganisms were present, but the LAB population was dominated by *Lp. plantarum* CRL 2211 since 94% of isolates corresponded to this strain (similarity greater than 87%), and only 6% showed fingerprints similar to that of *W. paramesenteroides* CRL 2182 (similarity greater than 99%) (Figure 2 and Figure S4). These results suggest that the fermentation of bean flour with the mixed starter was mainly conducted by the *Lp. plantarum* strain in a similar manner to that observed in chickpea doughs [19]. The robustness of *Lp. plantarum* starters during the propagation of sourdoughs and their dominance over the indigenous LAB microbiota has been reported [40,41]. This property is relevant for the successful application of a starter culture since its stability over time will ensure the reproducibility of the characteristics of the derived products [41].

### 3.6. In Silico Analyses

#### 3.6.1. Metabolic Modeling

**Figure 3:**
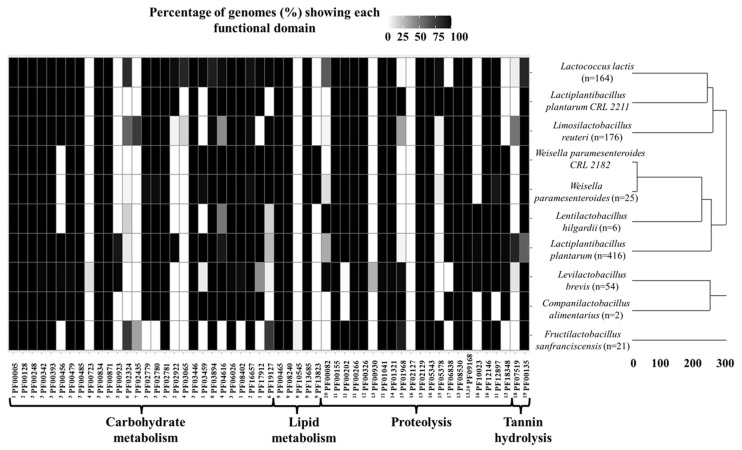
A heatmap showing the presence of different functional domains involved in sourdough fermentation (indicated as black boxes) in the genome of LAB present in alubia beans: ^1^ MsmK (oligosaccharide transport system), ^2^ Amyx (extracellular amylase), DexB (glucosidase hydrolyzing α(1-6)-linked gluco-oligosaccharides) and amylase, ^3^ the pentose phosphate pathway and phosphogluconate metabolism, ^4^ MalN and MalL (intracellular glucosyl hydrolases), ^5^ acetate kinase, ^6^ glucansucrase, ^7^ fructansucrase, ^8^ phosphoketolase, ^9^ Alcoholdehydrogenase, ^10^ proteinase prtP, ^11^ transaminase, ^12^ prolyl endopeptidase, ^13^ dipeptidyl-peptidase, ^14^ prolidase, ^15^ prolinase, ^16^ aminopeptidase, ^17^ methionine Υ-lyase, ^18^ tannase, ^19^ tannase-like domain. Similarities between LAB genomes are also illustrated in a dendrogram and expressed as distances between their characteristic functional domain profiles calculated by the complete linkage method (vertical axis). Sequence data were retrieved from the European Nucleotide Archive (ENA) at the European Bioinformatics Institute (EMBL-EBI) (https://www.ebi.ac.uk/ena/browser/search, accessed on 24 May 2024). The genome assemblies of *Lp. plantarum* CRL 2211 and *W. paramesenteroides* CRL 2182 were included.

Once the fermentation of alubia bean flours was carried out experimentally, two in silico analyses comprising metabolic and molecular modeling were performed for a better understanding of the fermentation process. For this purpose, functional domains associated with carbohydrate and lipid metabolism as well as proteolysis and tannin hydrolysis (Figure 3) were annotated in the genome sequences of *Lp. plantarum* CRL 2211, *W. paramesenteroides* CRL 2182 and other LAB that could be present in this substrate [16,22] (see Section 2.8.2). These metabolic functions play a major role in sourdough fermentation according to several authors [24,25,26]. With regard to carbohydrate metabolism, several domains comprising MsmK (oligosaccharide transport system), Amyx (extracellular amylase), DexB (glucosidase hydrolyzing α(1-6)-linked gluco-oligosaccharides), acetate kinase, phosphoketolase and other enzymes involved in the pentose phosphate and phosphogluconate pathway were observed in all species (Figure 3). Starch present in the dough is the main source of fermentable carbohydrates (such as maltodextrins, maltose and glucose) that are released during fermentation by amylase activities [25]. Similarly, intracellular glucosyl hydrolases MalN and MalL and glucansucrase were found in most species. In contrast, some specific activities include fructansucrase domains that were found only in the *Fr. sanfranciscensis* and *Lm. reuteri* genomes and may highlight potential synergistic metabolic interaction with other species lacking these functional domains.

On the other hand, alcohol dehydrogenase was the only functional domain involved in lipid metabolism that was annotated (Figure 3). This activity may play a relevant role in reducing the flavor-active (E)-2-nonenal and (E,E)-2,4-decadienal to the corresponding alcohols during growth in sourdough [25].

With regard to enzymes involved in proteolysis, aminopeptidase, dipeptidyl-peptidase, prolidase, prolyl endopeptidase and transaminase functional domains were ubiquitous in all LAB species. Several of these domains correspond to key enzymes in sulfur metabolism [24], together with prolinases and methionine Υ-lyases found in the *Lp. plantarum* CRL 2211 and *W. paramesenteroides* CRL 2182 strains and other LAB (Figure 3).

Finally, enzyme domains involved in ANF removal include prtP proteinase and tannase domains, which were annotated in most genomes. It should be noted that both the *Lp. plantarum* CRL 2211 and *W. paramesenteroides* CRL 2182 strains showed tannase-like domains. The presence of tannases in *Lp. plantarum* genomes was reported by Rha et al. [42]. The relevance of prtP proteinases and tannases in ANFs is discussed in the molecular modeling section (Section 3.6.2).

Once the functional domains of interest were annotated in the genomes of LAB involved in the fermentation of alubia beans, potential cross-feeding mechanisms between these species were elucidated. In this sense, essential symbionts and alternative symbionts were determined according to Belcour et al. [27]. Essential symbionts comprise key microorganisms present in every minimal community of LAB that are needed to fulfill one specific metabolic function such as the metabolism of each nutrient present in *Phaseolus vulgaris*. In contrast, alternative symbionts occur only in some of these communities.

Essential symbionts comprised seven genomes corresponding to *Lp. plantarum* (n = 3), *L. lactis* (n = 2), *Lm. reuteri* (n = 1) and *Lv. brevis* (n = 1). The number of genes associated with specific metabolic pathways involved in alubia bean flour fermentation ranged from 746 to 1107. These LAB species possess a higher number of metabolic and key functions associated with sourdough fermentation in agreement with previous studies [24,25,26], as well as genome annotation results (Figure 3). Regarding the strains inoculated to doughs, *W. paramesenteroides* CRL 2182 and *Lp. plantarum* CRL 2211 were essential and alternative symbionts with 755 and 1017 genes associated with specific metabolic pathways, respectively.

**Figure 4:**
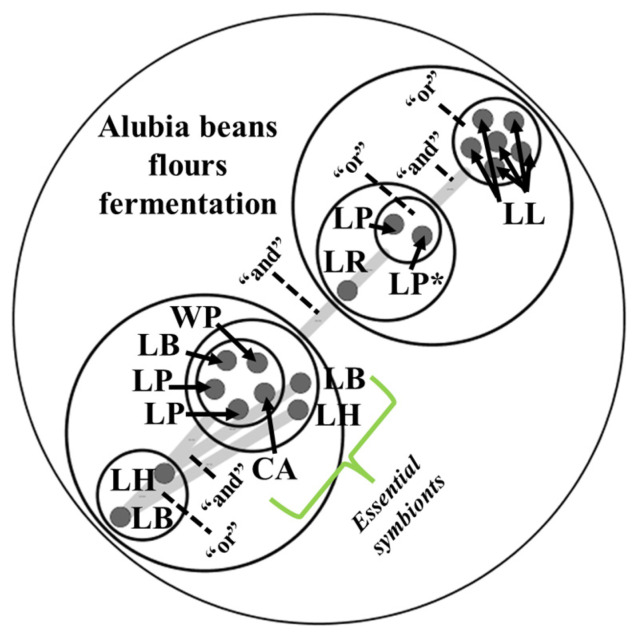
A metabolic network illustrating potential cross-feeding mechanisms between LAB present in alubia bean flours. Network nodes (i.e., circles containing different microbial communities showing equivalent metabolic functions) are connected by gray lines indicating synergistic relationships between communities and complementary metabolic functions. Metabolic function LAB from different nodes are needed to achieve the maximum number of end-products from alubia bean flours (this mutualistic relationship is indicated by the conjunction “and”), while LAB inside the same node play the same role and could be replaced by other members from the same community (this similar role is indicated by the conjunction “or”). CA: *Co. alimentarius*, LB: *Lv. brevis*, LH: *Ll. hilgardii*, LL: *L. lactis*, LP: *Lp. plantarum*, LP*: *Lp. plantarum* CRL 2211, LR: *Lm. reuteri*, WP: *W. paramesenteroides* CRL 2182.

To illustrate potential cross-feeding mechanisms, a total of 35 genomes comprising all essential symbionts (n = 7) and 11 alternative symbionts showing the highest genes associated with specific metabolic pathways (including *W. paramesenteroides* CRL 2182 and *Lp. plantarum* CRL 2211) were selected to generate a metabolic network. Figure 4 illustrates the synergistic mechanisms between all these LAB species and shows that some *Lv. brevis* and *Ll. hilgardii* as well as some *L. lactis* genomes display equivalent metabolic functions. Interestingly, potential cross-feeding between LAB species showing enzyme domains involved in ANF removal such as *Lp. plantarum* (Figure 3 and Figure 4) and other LAB species was observed. *Lp. plantarum* was present in different essential and alternative symbiotic microbial communities (Figure 4), and *W. paramesenteroides* CRL 2182, an essential symbiont, exerted potential cross-feeding interactions with *Lp. plantarum* CRL 2211. This behavior agrees with the experimental results described in this work where the co-inoculation of flours with both species contributed to higher ANF removal in sourdoughs. Mutualistic relationships between *Lp. plantarum* and other LAB like *W. paramesenteroides* in vegetable fermentation have been suggested by other authors [42], but few studies have reported symbiotic interactions between bacterial consortia in sourdough [43].

#### 3.6.2. Molecular Modeling of Phenolic Compound Release and Trypsin Inhibitor Hydrolysis

Once metabolic pathways and cross-feeding mechanisms that may be involved in alubia bean fermentation were explored, molecular modeling was performed. Simulations of tannin hydrolysis leading to phenolic compounds’ release (e.g., gallic acid) and trypsin inhibitors’ hydrolysis were carried out (Table S4). To ensure the quality of structures generated by homology, Ramachandran plots were generated (Figure S5), showing that Ramachandran outliers and Z-score values were below 0.05% and 2, respectively, and a number of favored residues higher than 98%.

**Figure 5:**
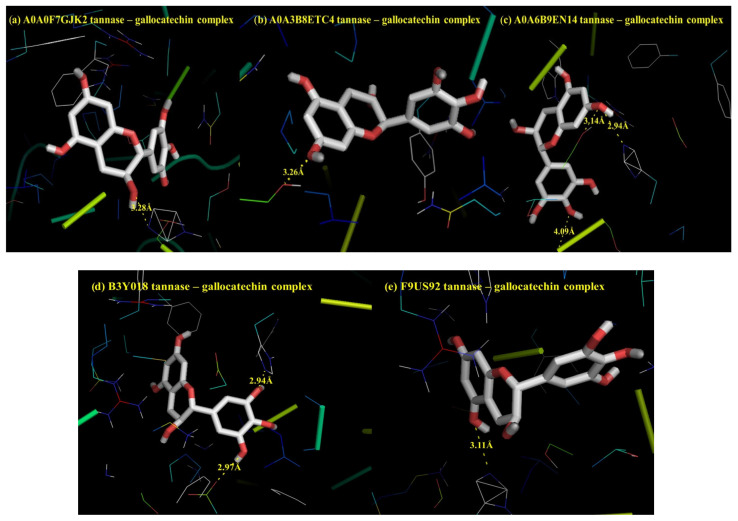
Potential interaction mechanisms, determined by molecular docking, between gallocatechin present in alubia bean flour and tannases from *Lp. plantarum* and *Lv. brevis*: (**a**) A0A0F7GJK2: residue HIS451 interacts with oxygen atoms from hydroxyl groups (OH) of gallocatechin (bond length 3.28 Å), (**b**) A0A3B8ETC4: residue SER117 interacts with oxygen atoms from OH of gallocatechin (bond length 3.26 Å), (**c**) A0A6B9EN14: residues HIS451, SER163 and GLY453 interact with oxygen atoms from OH of gallocatechin (bond lengths 2.94, 3.14 and 4.09 Å, respectively), (**d**) B3Y018: residues HIS451 and GLU357 interact with oxygen atoms from OH of gallocatechin (bond lengths 2.94 and 2.97 Å, respectively), (**e**) F9US92: residue HIS451 interacts with oxygen atoms from OH of gallocatechin (bond length 3.11 Å). Polar contacts between catalytic residues from active site and gallocatechin molecule as well as bond distance (Å) are indicated in yellow.

The affinity of gallocatechin and trypsin inhibitors to the active site of tannases and endopeptidases from *Lp. plantarum*, *Lv. brevis* and *L. lactis* was determined by molecular docking. As illustrated in Table S5, tannases from LAB present in alubia beans showed a relevant affinity for gallocatechin (−6.9 kcal/mol for F9US92 from *Lp. plantarum* and −5.4 kcal/mol for A0A3B8ETC4 from *Lv. brevis*). Figure 5 provides a graphical representation of interaction mechanisms between gallocatechin and LAB tannases. These mechanisms consist of polar contacts between catalytic residues from the active site and gallocatechin. Rha et al. [41] performed docking simulations of tannases from *Lp. plantarum* and reported similar interactions in the active site. Furthermore, the amino acid sequence analysis of bacterial, yeast and fungal tannases share an active site motif glycine and serine, a typical feature for serine hydrolase, in agreement with our results [43,44,45]. Docking affinity values might not correlate well with experimental binding energies, so molecular dynamic simulations of gallocatechin–tannase complexes and MM/PBSA binding energy calculations were performed for the two most relevant tannases: F9US92 and A0A3B8ETC4. Molecular dynamic simulations led to binding energy values of −11.3 kcal/mol and −5.9 kcal/mol for A0A3B8ETC4 and F9US92 when performed at 30 °C. These binding energies were higher than those reported by dos Santos et al. [46] for transmembrane proteins from *Staphylococcus aureus* and gallate complexes.

The affinity of trypsin inhibitors to LAB endopeptidases was investigated through protein–protein docking (Table S6). The Q49SH0 protease from *L. lactis* showed slightly higher relative affinities to Bowman–Birk-type proteinase inhibitors P81483 and P81484 than the B7VFD1 peptidase from *Lp. plantarum*. However, when the binding energy was determined using a more accurate approach based on molecular dynamics following the MM/GBSA method, B7VFD1 led to higher energies (−49.5 kcal/mol). Both proteases interacted with inhibitors through salt bridges, electrostatic interactions and short contacts (Table S7). Potential molecular interactions agree with those previously elucidated for the Kunitz-type trypsin inhibitor from *Tamarindus indica* L and trypsin complexes [47], but binding energies were higher than those reported for the Kunitz-type protease inhibitor of other legumes like *Mucuna pruriens* complexed to trypsin [48].

To validate the docking protocol, gallocatechin was redocked to the active sites of all tannases in triplicate (Figure S6). Trypsin inhibitors were also redocked to endopeptidases. The Root Mean Squared Deviations (RMSDs) obtained for gallocatechin and trypsin inhibitors were lower than 0.5 Å.

The molecular modeling performed provides information on the possible modes of action of enzyme domains present in LAB genomes that, according to experimental data, play an important role in the fermentation of alubia beans.

### 3.7. Comparison of Processing Methods for Improving Nutri-Functional Properties of Beans

Proper processing is required prior to legume consumption in order to improve their biological value. Soaking, cooking and germination are traditional processing methods with different results on the nutritional quality of legumes, depending on the grains they are applied to, including some negative impacts on vitamin and mineral content. Therefore, fermentation with both *Lp. plantarum* CRL 2211 and *W. paramesenteroides* CRL 2182 was compared with other methods in order to define the best processing strategy. In untreated flour (control), trypsin, α-chymotrypsin, α-amylase inhibitor and tannin contents were 2.9 ± 0.8 TIA/g; 247.0 ± 44.0 CUI/g; 701.0 ± 49.9 AUI/g and 7.0 ± 0.7 mg GAE/100 g, respectively (Table 2), significantly higher than the observed in chickpea flours [19]. Boiling was the most efficient method for the complete elimination of trypsin and α-amylase inhibitors. Biological methods (germination and fermentation) were also appropriate for removing enzyme inhibitors and were even more efficient than soaking and microwaving for decreasing trypsin, α-chymotrypsin and α-amylase inhibitors (Table 2), whereas fermentation was the best method for decreasing total tannins (from 7.0 ± 0.0 to 1.3 ± 0.6 mg GAE/100 g). Fermentation with both LAB strains was able to remove 79.31% of TIA, 92.71% of CUI, 91.44% of AUI and 81.43% of tannins. When applied to chickpea flours, a similar removal of tannins was observed (approx. 80%), whereas trypsin, chymotrypsin and amylase inhibitors were removed significantly less (approx. 65%) [19].

FAA concentration from raw and treated flours was analyzed as the reference of the impact of processing on relevant nutrients (Table 2, Figure S7). Biological methods, such as fermentation and germination, increased by 50% and 20% total FAA content (from 521.18 mg/kg to 779.94 mg/kg and 611.82 mg/kg), respectively, whereas cooking methods, such as boiling and microwaving, produced 20% and 10% losses of FAAs (from 521.18 mg/kg to 415 mg/kg and 463.86 mg/kg), respectively. Fermentation with the selected strains highly released glutamic acid, glutamine and essential amino acids like arginine, leucine and lysine, almost doubling their concentrations in the doughs. Curiel et al. [35] reported that the fermentation of Italian legumes by a starter mixture composed of *Lp. plantarum* C48 and *Lv. brevis* AM7 produced an average increase in FAA concentrations of 23–28% for kidney beans, grass pea, chickpea and lentil sourdoughs, whereas Coda et al. [7] observed that the fermentation of faba bean matrices with *Lp. plantarum* VTT E-133328 caused a marked increase in total FAAs (from 2 up to 3.5 times, compared to unfermented flours). It is worth mentioning that fermentation did not negatively affect other nutritional components relevant to health such as dietary fiber since a decrease of less than 5% was observed in the fermented flour (unpublished results).

Regarding functional properties, fermentation and germination increased the TPC of bean flours (from 466.7 ± 33.0 up to 745.7 ± 24.9 and 696.2 ± 45.5, respectively), whereas cooking treatments decreased their content. Accordingly, the antioxidant potential measured by free radical scavenging capacities was greatly increased by these biological methods (Table 2). The enrichment in phenolics and antioxidant activity was also reported for other legume flours and milk fermented with the *Lp. plantarum*, *Lp. pentosus*, *Lv. brevis*, *L. lactis* subsp. *lactis* and *Pediococcus* strains [8,35,49]. The improvement in the antioxidant properties of food depends on the enzyme activities of the starter used for fermentation [5]. In this regard, LAB display a wide portfolio of enzymes (hydrolases, decarboxylases and reductases) involved in the metabolism of the phenolics of plants for energy generation and/or detoxification [25,50].

In view of these results, the modification of polyphenols was particularly determined after fermentation. Twenty-two phenolic compounds were identified in non-fermented kidney bean flour. Among them, phenolic acids were the major group found, representing more than 80% of the total content. The hydroxycinnamic acid derivatives identified were ferulic, caffeic, chlorogenic, p-cinnamic and p-coumaric acids, whereas hydroxybenzoic acid derivatives were represented by gallic, syringic, protocatechuic and vanillic acids.

Among flavonoids, the main compounds found were quercetin-3-glucoside, rutin, daidzein, genistein, resveratrol, naringenin, kaempferol and herspertin. After fermentation (both spontaneous and LAB started), qualitative and quantitative differences in the identified phenolic compounds were observed (Table 3). Fermentation with both *Lp. plantarum* CRL 2211 and *W paramesenteroides* CRL 2182 increased more than two-fold (up to 245 mg/kg TPC) and modified the polyphenol profile by increasing gallic acid and decreasing caffeic, ferulic and vanillic acids and quercetin-3-glucoside. The increase in hydroxybenzoic acids could be due to gallotannin hydrolysis by esterases, whereas the decrease in total hydroxycinnamic acids could be the result of metabolism by LAB decarboxylases and reductases. Phenolic acid metabolism by LAB has been reported for the *Lactiplantibacillus*, *Levilactobacillus* and *Furfurilactobacillus* genera as well as *Lm. fermentum* which are present in cereal, legume and vegetable fermentations [50]. Modifications in the polyphenol profile were reported with some differences for kidney beans fermented in the solid and liquid state by the *B. subtilis* or *Lp. plantarum* strains [9].

## 4. Conclusions

Over the past decade, there has been a notable increase in the production of beans on a global scale, driven by the rising demand for plant-based foods. Consequently, novel consumption options have been proposed for these pulses, including foods derived from fermented legumes. Fermentation can enhance the functional properties of legumes; however, the appropriate selection of microorganisms is crucial to ensure the optimal biotransformation of the target metabolites. In the present study, two bacterial strains, *Lp. plantarum* CRL2211 and *W. paramesenteroides* CRL2182, isolated from Argentinian pulses were able to grow and ferment bean flour, with *L. plantarum* being particularly dominant. The fermentation of bean flour with the mixed starter resulted in a significant increase in TPC and FAAs and a decrease in ANFs when fermented at 37 °C for 24 h. Metabolic modeling highlighted the presence of key enzyme domains involved in proteolysis and tannin hydrolysis in the genome sequences of both *Lp. plantarum* CRL 2211 and *W. paramesenteroides* CRL 2182. These metabolic activities of interest play a significant role in the reduction in ANFs in bean flours in agreement with the experimental results. Furthermore, metabolic modeling revealed potential symbiotic interactions between these two strains and other LAB that may be present in fermented bean flour. Molecular modeling elucidated the potential mechanisms of action of the tannase and endopeptidase enzyme domains identified in the *Lp. plantarum* CRL 2211 and *W. paramesenteroides* CRL 2182 genomes. Therefore, computational models corroborate the experimental evidence, highlighting the potential applications of *Lp. plantarum* CRL2211 and *W. paramesenteroides* CRL2182 as starter cultures to enhance the nutri-functional quality of fermented bean flour.

Our study illustrates that experimental and advanced computational tools can offer valuable complementary insights, contributing to a more comprehensive understanding of the biological modification of a food matrix. These results could be of great interest in the development of functional foods.

## Figures and Tables

**Table 1:** Treatment combinations and the experimental (Exp) and delta (Δ, difference between fermented and raw flour) data of the responses. A: *Lp. plantarum* CRL 2211, B: *W. paramesenteroides* CRL 2182, C: temperature (°C), D: time (h), E: dough yield (%), LAB: lactic acid bacteria, TPC: total phenolic content, GAE: gallic acid equivalents.

Fermentation Conditions						LAB (log CFU/g)		pH		TPC (mg GAE/100 g)		Tannin Content (mg GAE/100 g)		Trypsin Inhibitors (mg/g)	
Variables															
RUN	A	B	C	D	E	Exp	Δ	Exp	Δ	Exp	Δ	Exp	Δ	Exp	Δ
1	0	0	37	24	160	3.40	3.00	6.34	0.06	291.96	−13.04	41.79	2.21	3.64	0.06
2	1	1	37	24	160	8.64	8.24	4.24	2.16	382.14	77.14	12.23	31.77	1.53	2.17
3	0	1	30	24	200	8.40	8.00	4.17	2.03	322.32	77.32	10.18	22.32	0.22	2.03
4	0	1	30	8	160	7.93	7.53	4.92	1.48	330.36	25.36	23.04	20.96	1.52	2.18
5	1	1	37	8	200	8.41	8.01	4.27	1.93	319.64	74.64	14.73	17.77	0.62	1.63
6	1	0	30	8	160	8.48	8.08	4.77	1.63	329.46	24.46	26.61	17.39	2.34	1.36
7	0	0	37	8	200	6.00	5.60	6.10	0.10	157.14	−87.86	29.91	2.59	2.15	0.10
8	1	0	30	24	200	8.74	8.34	3.75	2.45	333.04	88.04	3.39	29.11	0.10	2.15
9	0	1	30	8	200	7.94	7.54	4.59	1.61	300.00	55.00	17.05	15.45	0.64	1.61
10	1	1	37	24	200	8.96	8.56	3.81	2.39	348.21	103.21	0.00	32.50	2.00	2.25
11	1	1	37	8	160	8.24	7.84	4.87	1.53	349.11	44.11	21.16	22.84	2.24	1.46
12	0	0	37	8	160	1.88	1.48	6.33	0.07	258.93	−46.07	42.14	1.86	3.63	0.07
13	0	0	37	24	200	5.50	5.10	4.96	1.24	267.86	22.86	25.63	6.87	1.93	0.32
14	0	1	30	24	160	8.32	7.92	4.21	2.19	247.32	−57.68	33.66	10.34	1.72	1.98
15	1	0	30	24	160	8.65	8.25	4.22	2.18	377.68	72.68	6.07	37.93	2.07	1.63
16	1	0	30	8	200	8.32	7.92	4.24	1.96	320.54	75.54	16.07	16.43	0.69	1.56
17	0	1	37	24	160	8.46	8.06	4.58	1.82	341.96	36.96	15.63	28.37	2.12	1.58
18	0	1	37	8	200	8.30	7.90	4.63	1.57	277.68	32.68	23.13	9.37	0.88	1.37
19	1	1	30	8	160	8.33	7.93	4.56	1.84	353.00	48.00	30.18	13.82	2.46	1.24
20	0	0	30	8	160	4.50	4.10	6.02	0.38	289.29	−15.71	43.48	0.52	3.32	0.38
21	1	0	37	24	160	8.44	8.04	4.24	2.16	349.11	44.11	6.61	37.39	1.93	1.77
22	1	1	30	24	200	8.93	8.53	4.06	2.14	347.32	102.32	0.00	32.50	0.36	1.89
23	1	0	37	8	200	8.67	8.27	4.41	1.79	306.25	61.25	17.23	15.27	0.46	1.79
24	0	0	30	24	200	5.71	5.31	5.30	0.90	250.00	5.00	30.36	2.14	1.01	1.24
25	1	0	37	8	160	7.61	7.21	4.78	1.62	333.93	28.93	32.14	11.86	2.08	1.62
26	0	1	37	8	160	8.18	7.78	4.72	1.68	318.75	13.75	34.91	9.09	2.28	1.42
27	0	0	30	8	200	5.40	5.00	5.78	0.42	243.75	−1.25	30.98	1.52	1.35	0.90
28	1	1	30	24	160	8.70	8.30	4.17	2.23	359.82	54.82	8.39	35.61	1.57	2.13
29	0	1	37	24	200	8.40	8.00	4.26	1.94	317.86	72.86	4.11	28.39	0.31	1.94
30	1	0	37	24	200	9.64	9.24	3.92	2.28	307.14	62.14	0.00	32.50	0.00	2.25
31	1	1	30	8	200	8.34	7.94	4.26	1.94	269.64	24.64	13.48	19.02	0.71	1.54
32	0	0	30	24	160	4.47	4.07	4.93	1.47	312.50	7.50	40.09	3.91	2.23	1.47

**Table 2:** Effect of processing on FAN removal, and functional properties in alubia bean flours. Means of same row with different lowercase letters show significant difference (*p* < 0.05). * Bean flours were fermented with *Lp. plantarum* CRL 2211 and *W. paramesenteroides* CRL 2182.

	Control	Fermentation *	Germination	Soaking	Cooking	Microwave
Antinutritional factors						
Trypsin inhibitors (TIA mg/g)	2.9 ± 0.8 ^a^	0.6 ± 0.4 ^b^	1.0 ± 0.6 ^b^	2.0 ± 0.5 ^b,c^	0.0 ± 0.0 ^d^	1.9 ± 0.1 ^e^
α-Chymotrypsin inhibitors (CUI/g)	247.0 ± 44.0 ^a^	18.0 ± 15.0 ^b^	44.0 ± 30.0 ^b,c^	124.0 ± 85.0 ^c^	12.0 ± 10.0 ^b^	70.0 ± 27.0 ^c^
α-Amylase inhibitors (AUI/g)	701.0 ± 49.0 ^a^	60.0 ± 35.0 ^b^	286.0 ± 69.0 ^c^	514.0 ± 85.0 ^a,d^	0.0 ± 0.0 ^e^	325.0 ± 125.0 ^c^
Tannins (mg GAE/100 g)	7.0 ± 0.7 ^a^	1.3 ± 0.6 ^b^	4.6 ± 1.1 ^a,c^	5.8 ± 0.8 ^a,c^	5.3 ± 1.1 ^c^	6.5 ± 0.9 ^a^
Main amino acids content (mg/kg)						
Glutamic acid	25.4 ± 1.2 ^a^	61.6 ± 2.3 ^b^	36.7 ± 2.4 ^c^	36.2 ± 3.1 ^c^	44.6 ± 2.3 ^d^	40.0 ± 3.5 ^d^
Glutamine	147.2 ± 4.1 ^a^	281.9 ± 5.5 ^b^	134.8 ± 6.5 ^c^	14.9 ± 3.4 ^d^	162.8 ± 2.6 ^e^	203.6 ± 4.3 ^f^
Arginine	131.2 ± 4.8 ^a^	207.3 ± 5.8 ^b^	166.4 ± 4.1 ^c^	28.0 ± 2.8 ^d^	68.2 ± 3.3 ^e^	104.7 ± 6.1 ^f^
Leucine	18.6 ± 2.4 ^a^	35.7 ± 3.6 ^b^	2.2 ± 1.2 ^c^	11.3 ± 1.9 ^d^	1.5 ± 1.0 ^c,e^	1.6 ± 1.3 ^c,e^
Lysine	6.4 ± 1.8 ^a^	41.8 ± 3.3 ^b^	22.6 ± 3.7 ^c^	0.00 ± 0.0 ^d^	9.2 ± 2.4 ^a,e^	8.1 ± 2.1 ^a,e^
Functional properties						
Total phenols (mg GAE/100 g)	466.7 ± 33.0 ^a,b^	745.7 ± 24.9 ^c^	696.2 ± 45.5 ^c^	482.9 ± 52.5 ^a^	354.3 ± 71.0 ^b,d^	302.9 ± 23.4 ^d^
DPPH antioxidant activity (%)	35.0	71.0	65.0	36.0	23.0	33.0

**Table 3:** Main polyphenols detected on bean flour extracts: Raw flour, spontaneously fermented flour (no starter, 37 °C, 24 h) and LAB-fermented flour (*Lp. plantarum* CRL 2211 + *W. paramesenteroides* CRL 2182, 37 °C, 24 h). Means of same row with different lowercase letters show significant difference (*p* < 0.05).

Phenolic Compounds	Compound (mg/kg)	Raw Flour	Spontaneous Fermentation	LAB Fermentation
Hydroxycinnamic acids	Caffeic acid	12.83 ± 0.77 ^a^	4.69 ± 1.70 ^c^	7.62 ± 0.61 ^b^
	Chlorogenic acid	4.06 ± 0.56 ^a^	1.11 ± 0.63 ^c^	10.11 ± 0.44 ^b^
	p-cinnamic acid	5.80 ± 1.35 ^a^	6.19 ± 2.47 ^a^	6.30 ± 1.44 ^a^
	p-coumaric acid	13.12 ± 1.16 ^a^	2.83 ± 1.33 ^c^	27.64 ± 0.89 ^b^
	Ferulic acid	41.60 ± 2.89 ^a^	5.16 ± 2.49 ^b^	4.09 ± 1.67 ^b^
Hydroxybenzoic acids	Gallic acid	1.90 ± 1.70 ^a^	68.55 ± 11.45 ^c^	177.31 ± 3.64 ^b^
	Protocatechuic acid	1.78 ± 0.48 ^a^	1.22 ± 0.78 ^a,b^	0.54 ± 0.44 ^b^
	Syringic acid	0.45 ± 0.40 ^a^	0.24 ± 0.24 ^a^	0.46 ± 0.46 ^a^
	Vanillic acid	13.96 ± 2.76 ^a^	3.56 ± 3.12 ^b,c^	8.87 ± 2.69 ^b^
Flavonoids	Daidzein	0.14 ± 0.08 ^a^	0.13 ± 0.11 ^a,b^	0.18 ± 0.10 ^a^
	Genistein	0.31 ± 0.25 ^a^	0.27 ± 0.30 ^a,b^	0.33 ± 0.29 ^a^
	Hesperetin	0.05 ± 0.05 ^a^	0.04 ± 0.05 ^a^	0.08 ± 0.05 ^a,b^
	Kaempferol	0.04 ± 0.03 ^a^	0.06 ± 0.15 ^b^	0.08 ± 0.11 ^b^
	Naringenin	0.28 ± 0.18 ^a^	0.19 ± 0.19 ^a^	0.44 ± 0.21 ^a,b^
	Quercetin-3-glucoside	2.22 ± 0.55 ^a^	0.43 ± 0.40 ^b^	0.50 ± 0.50 ^b^
	Resveratrol	0.10 ± 0.10 ^a^	0.17 ± 0.15 ^a,b^	0.18 ± 0.11 ^a,b^
	Rutin	1.18 ± 0.66 ^a^	1.01 ± 0.83 ^a,b^	0.98 ± 0.61 ^b^
Total amount *		≅99.82	≅95.83	≅245.93

* Apigenin, quercetin-3-galactoside, luteolin and phloretin were in traces and omitted.

## Data Availability

The original contributions presented in the study are included in the article and Supplementary Materials, further inquiries can be directed to the corresponding author.

## Supplementary Materials

The following supporting information can be downloaded at https://www.mdpi.com/article/10.3390/foods13132105/s1, Table S1: Selected factors and value levels used for bean flour fermentation. Table S2: Main macronutrient components of alubia bean flour. Table S3: Validation data for determination of phenolic compounds in bean flours. Table S4: Microbial serine-type endopeptidase and tannases from *Lp. plantarum*, *L. lactis* and *Lv. brevis* and Bowman–Birk-type proteinase inhibitors used to simulate enzyme–substrate interactions in tannin hydrolysis and phenol release as well as the removal of trypsin inhibitors during flour fermentation. Table S5: Affinity and relative affinity values determined by molecular docking of tannases from *Lp. plantarum* and *Lv. brevis* to gallocatechin. Table S6: Docking scores and relative affinity values determined by molecular docking of serine-type endopeptidase from *Lp. plantarum* (B7VFD1) and *L. lactis* (Q49SH0) to P81483 and P81484 Bowman–Birk-type proteinase inhibitors. Table S7: Interaction mechanisms and bonds determined by molecular docking of serine-type endopeptidase from *Lp. plantarum* (B7VFD1) and *L. lactis* (Q49SH0) to P81483 and P81484 Bowman–Birk-type proteinase inhibitors. Figure S1: Growth curves of LAB strains in sterile bean extracts. Figure S2: Correlations between experimental and response variables in alubia bean fermentation. Figure S3: Pareto charts showing the effects of alubia bean fermentation conditions on the different responses. Figure S4: Dendrogram obtained by cluster analysis rep-PCR fingerprints of colonies isolated from fermented alubia bean flours with selected starter. Figure S5: Ramachandran plots from homology models of serine-type endopeptidase and tannases from *Lp. plantarum*, *L. lactis* and *Lv. brevis* and Bowman–Birk-type proteinase inhibitors. Figure S6: Redocking of gallocatechin present in alubia bean flour and tannases from *Lp. plantarum* and *Lv. brevis*. Figure S7: Concentration of FAAs in alubia bean flours after different processing methods.
