# Supplementary material for: Fermentation with Lactic Acid Bacteria for Bean Flour Improvement: Experimental Study and Molecular Modeling as Complementary Tools
Source: Foods. 2024 Jul 2;13(13):2105. doi: 10.3390/foods13132105 (PMC11241767; doi:10.3390/foods13132105)
Supplement: Supplementary file 1 [file foods-13-02105-s001.zip › foods-3058131-supplementary.pdf]

Fermentation with Lactic Acid Bacteria for Bean Flour Improvement: Experimental Study and Molecular Modeling as Complementary Tools

Carlos Sabater [1,2,†], Gabriel D. Sáez [3,4,†], Nadia Suárez [3], Marisa S. Garro [3], Abelardo Margolles [1,2] and Gabriela Zárate [3,4,*]

[1] Department of Microbiology and Biochemistry of Dairy Products, Dairy Research Institute of Asturias (IPLA), Spanish National Research Council (CSIC), Paseo Río Linares S/N, 33300 Villaviciosa, Asturias, Spain; carlos.sabater@csic.es (C.S.); amargolles@ipla.csic.es (A.M.)
[2] Health Research Institute of Asturias (ISPA), 33011 Oviedo, Asturias, Spain
[3] Laboratory of Technological Ecophysiology, Reference Centre for Lactobacilli (CERELA-CONICET), Chacabuco 145, San Miguel de Tucumán 4000, Argentina; gabrieldsaez@yahoo.com.ar (G.D.S.); nsuarez@cerela.org.ar (N.S.); mgarro@cerela.org.ar (M.S.G.)
[4] Department of Food Microbiology, University of San Pablo Tucumán, Av. Solano Vera y Camino a Villa Nougués, San Pablo 4129, Tucumán, Argentina
* Correspondence: gzarate@cerela.org.ar
† These authors contributed equally to this work.

Table S1. Selected factors and value levels used as conditions for fermentation of alubia beans flours.

Code	Factors	Level -1	Level +1
A	*Lactiplantibacillus plantarum* CRL 2211 (Log CFU/g)	0	7
B	*Weissella paramesenteroides* CRL 2182 (Log CFU/g)	0	7
C	Temperature (°C)	30	37
D	Time (h)	8	24
E	Dough yield (DY)	160	200

Table S2. Main macronutrient components of alubia beans flour.

Carbohydrates (%)		Fat (%) Total lipids		Fat (%) Fatty acids		Proteins (Essential aminoacids)	
Starch	76.5	Triglycerides	39.0	Palmitic acid	13.9	Histidine	5.2
Monosaccharides	10.2	Diglycerides	12.5	Estearic acid	2.4	Leucine	18.6
Sucrose	3.1	Monoglycerides	2.6	Oleic acid	5.1	Isoleucine	8.7
Raffinose	1.2	Sterols	8.5	*cis*-vaccenic acid	1.8	Valine	11.7
Stachyose	4.2	Phospholipids	19.9	Linoleic acid	21.8	Threonine	7.8
Verbascose	0.6	Ceramide and glucosylceramide	1.2	Linolenic acid	54.9	Phenylalanine	9.2
						Tryptophan	8.5
		Other unknown lipids	16.1			Lysine	6.4

Table S3. Retention times (min), calibration equations, correlation coefficients, sensitivity (µg/Kg) and repeatability (%) of the monitored compounds.

Compound	Retention Time (min)	Linear equations	R^2	Sensitivity	
				LOD µg/Kg	LOQ µg/Kg
**Gallic acid**	1.7	y = 4,481,700x	0.9996	0.49	1.63
**Protocatechuic acid**	2.9	y = 2,010,835x	0.9999	0.12	0.40
**Chlorogenic acid**	4.2	y = 4,996,159x	0.9991	0.12	0.36
**Vanillic acid**	5.0	y = 449,059x	0.9996	0.75	2.47
**Caffeic acid**	5.1	y = 6,055,373x	0.9996	0.14	0.45
**Syringic acid**	5.3	y = 271,734x	0.9982	0.33	1.08
**p-Coumaric acid**	6.9	y = 5,646,083x	0.9999	0.23	0.75
**Ferulic acid**	7.7	y = 1,995,261x	0.9991	0.19	0.63
**Rutin**	7.7	y = 4,882,345x	0.9999	0.19	0.64
**Quercetin-3-galactoside**	8.0	y = 8,923,578x	0.9991	0.29	0.97
**Quercetin-3-glucoside**	8.2	y = 4,360,387x	0.9999	0.15	0.51
**Resveratrol**	10.8	y = 2,229,418x	0.9998	0.05	0.17
**Daidzein**	11.1	y = 1,709,516x	0.9965	0.02	0.07
**Luteolin**	11.4	y = 6,622,982x	0.9989	0.04	0.13
**Quercetin**	11.5	y = 8,768,515x	0.9979	0.03	0.10
**Cinnamic acid**	11.7	y = 1,240,614x	0.9995	0.31	1.03
**Phloretin**	12.0	y = 25,489,951x	0.9999	0.01	0.03
**Naringenin**	12.05	y = 6,140,859x	0.9984	0.05	0.16
**Apigenin**	12.1	y = 5,146,699x	0.9994	0.03	0.11
**Genistein**	12.1	y = 1,299,352x	0.9962	0.07	0.22
**Kaempferol**	12.2	y = 318,331x	0.9996	0.17	0.05
**Hesperetin**	12.3	y = 4,828,599x	0.9981	0.01	0.03

Table S4. Microbial serine-type endopeptidase and tannases from *Lactiplantibacillus plantarum*, *Lactococcus lactis* and *Levilactobacillus brevis* and Bowman-Birk type proteinase inhibitors collected from UNIPROT (https://www.uniprot.org/ last accessed: 24/05/2024) used to simulate enzyme-substrate interactions in tannin hydrolysis and phenol release as well as the removal of trypsin inhibitors during flour fermentation.

Compounds studied	Enzyme (UNIPROT code)	Activity	Species	Structure source
**Trypsin inhibitors**	P81483	Bowman-Birk type proteinase inhibitor PVI-4	Bean (*Phaseolus vulgaris*)	Homology modelling
	P81484	Bowman-Birk type proteinase inhibitor PVI-3	Bean (*Phaseolus vulgaris*)	Homology modelling
**Microbial peptidases**	B7VFD1	Serine-type endopeptidase (prtP family of subtilases)	*Lactiplantibacillus plantarum*	Homology modelling
	Q49SH0	Serine-type endopeptidase (prtP family of subtilases)	*Lactococcus lactis*	Homology modelling
**Microbial tannases**	A0A0F7GJK2	Tannase	*Lactiplantibacillus plantarum*	Homology modelling
	A0A3B8ETC4	Tannase	*Levilactobacillus brevis*	Homology modelling
	A0A6B9EN14	Tannase	*Lactiplantibacillus plantarum*	Homology modelling
	B3Y018	Tannase	*Lactiplantibacillus plantarum*	Cristallized structure
	F9US92	Tannase	*Lactiplantibacillus plantarum*	Homology modelling

Table S5. Affinity (Kcal/mol; mean, and standard deviation of top 9 docking poses, SD), and relative affinity (expressed as percentage, %) values determined by molecular docking of tannases from *Lactiplantibacillus plantarum* and *Levilactobacillus brevis* to gallocatechin. [a,b]Statistically significant (p < 0.05) differences considering the scoring function of Autodock vina software (2-3 Kcal/mol).

		**Gallocatechin**		
**Enzyme (UNIPROT code)**	**Species**	**Affinity Mean (Kcal/mol)**	**Affinity SD (Kcal/mol)**	**Relative affinity (%)**
A0A0F7GJK2	*Lactiplantibacillus plantarum*	-6.3[a]	0.5	91.7
A0A3B8ETC4	*Levilactobacillus brevis*	-5.4[a]	0.3	78.8
A0A6B9EN14	*Lactiplantibacillus plantarum*	-6.8[a]	0.2	98.9
B3Y018	*Lactiplantibacillus plantarum*	-6.5[a]	0.2	95.0
**F9US92**	***Lactiplantibacillus plantarum***	**-6.9[a]**	**0.2**	**100.0**

Table S6. Docking scores (mean, and standard deviation of top 10 docking poses, SD), and relative affinity (expressed as percentage, %) values determined by molecular docking of Serine-type endopeptidase from *Lactiplantibacillus plantarum* (B7VFD1) and *Lactococcus lactis* (Q49SH0) to P81483 and P81484 Bowman-Birk type proteinase inhibitors. [a,b]Statistically significant ($p < 0.05$) differences between enzymes.

Enzyme (UNIPROT code)	Species	Docking score	Docking score SD	Relative affinity (%)
		**P81483**		
B7VFD1	*Lactiplantibacillus plantarum*	-157.0[a]	4.1	97.9
Q49SH0	*Lactococcus lactis*	-160.4[a]	6.7	100.0
		**P81484**		
B7VFD1	*Lactiplantibacillus plantarum*	-157.0[a]	4.1	97.9
Q49SH0	*Lactococcus lactis*	-160.4[a]	6.7	100.0

Table S7. Interaction mechanisms and bonds determined by molecular docking of Serine-type endopeptidase from *Lactiplantibacillus plantarum* (B7VFD1) and *Lactococcus lactis* (Q49SH0) to P81483 and P81484 Bowman-Birk type proteinase inhibitors. Interactions between aminoacid residues are shown. Res Num: residue number, Res Name: residue name.

**B7VFD1 protease - P81483 inhibitor complex**				
**Potential Hydrogen Bonds**				
**RESIDUE-1**		**RESIDUE-2**		**Distance**
**Res Num**	**Res Name**	**Res Num**	**Res Name**	**(Å)**
68	THR	145	ASN	2.96
**Potential Salt Bridges**				
**RESIDUE-1**		**RESIDUE-2**		**Distance**
**Res Num**	**Res Name**	**Res Num**	**Res Name**	**(Å)**
37	ARG	159	ASP	3.29
37	ARG	159	ASP	2.83
37	ARG	159	ASP	3.03
37	ARG	159	ASP	3.90
**Potential Favorable Electrostatic Interactions**				
**RESIDUE-1**		**RESIDUE-2**		**Distance**
**Res Num**	**Res Name**	**Res Num**	**Res Name**	**(Å)**
30	LYS	165	ASP	2.89
30	LYS	167	GLU	9.28
**Potential Short Contacts**				
**RESIDUE-1**		**RESIDUE-2**		**Distance**
**Res Num**	**Res Name**	**Res Num**	**Res Name**	**(Å)**
30	LYS	165	ASP	2.89
30	LYS	165	ASP	2.70
35	GLN	169	VAL	2.73
37	ARG	161	TYR	2.51
68	THR	169	VAL	2.67
69	ASN	145	ASN	2.17

**Q49SH0 protease - P81483 inhibitor complex**				
**Potential Hydrophobic Interactions**				
**RESIDUE-1**		**RESIDUE-2**		**Distance**
**Res Num**	**Res Name**	**Res Num**	**Res Name**	**(Å)**
554	ALA	43	LEU	4.01
**Potential Salt Bridges**				
**RESIDUE-1**		**RESIDUE-2**		**Distance**
**Res Num**	**Res Name**	**Res Num**	**Res Name**	**(Å)**
550	ASP	42	ARG	3.81
**Potential Favorable Electrostatic Interactions**				
**RESIDUE-1**		**RESIDUE-2**		**Distance**
**Res Num**	**Res Name**	**Res Num**	**Res Name**	**(Å)**
520	ASP	25	HIS	5.77
550	ASP	42	ARG	6.16
**Potential Unfavorable Electrostatic Interactions**				
**RESIDUE-1**		**RESIDUE-2**		**Distance**
**Res Num**	**Res Name**	**Res Num**	**Res Name**	**(Å)**
493	LYS	25	HIS	9.32
**Potential Short Contacts**				
**RESIDUE-1**		**RESIDUE-2**		**Distance**
**Res Num**	**Res Name**	**Res Num**	**Res Name**	**(Å)**
436	ASP	59	ILE	2.22
440	LEU	58	SER	2.65
519	THR	25	HIS	2.22
546	GLN	24	ASN	2.60
547	LYS	64	ILE	2.43
554	ALA	43	LEU	2.94
554	ALA	43	LEU	2.88
554	ALA	43	LEU	1.70

Table S7. Cont.

B7VFD1 protease - P81484 inhibitor complex				
**Potential Hydrogen Bonds**				
**RESIDUE-1**		**RESIDUE-2**		**Distance**
**Res Num**	**Res Name**	**Res Num**	**Res Name**	**(Å)**
67	THR	145	ASN	2.96
**Potential Salt Bridges**				
**RESIDUE-1**		**RESIDUE-2**		**Distance**
**Res Num**	**Res Name**	**Res Num**	**Res Name**	**(Å)**
36	ARG	159	ASP	3.29
36	ARG	159	ASP	2.83
36	ARG	159	ASP	3.03
36	ARG	159	ASP	3.90
**Potential Favorable Electrostatic Interactions**				
**RESIDUE-1**		**RESIDUE-2**		**Distance**
**Res Num**	**Res Name**	**Res Num**	**Res Name**	**(Å)**
29	LYS	165	ASP	2.89
29	LYS	167	GLU	9.28
**Potential Short Contacts**				
**RESIDUE-1**		**RESIDUE-2**		**Distance**
**Res Num**	**Res Name**	**Res Num**	**Res Name**	**(Å)**
29	LYS	165	ASP	2.89
29	LYS	165	ASP	2.70
34	GLN	169	VAL	2.73
36	ARG	161	TYR	2.51
67	THR	169	VAL	2.67
68	ASN	145	ASN	2.17

Q49SH0 protease - P81484 inhibitor complex				
**Potential Hydrophobic Interactions**				
**RESIDUE-1**		**RESIDUE-2**		**Distance**
**Res Num**	**Res Name**	**Res Num**	**Res Name**	**(Å)**
554	ALA	42	LEU	4.01
**Potential Salt Bridges**				
**RESIDUE-1**		**RESIDUE-2**		**Distance**
**Res Num**	**Res Name**	**Res Num**	**Res Name**	**(Å)**
550	ASP	41	ARG	3.81
**Potential Favorable Electrostatic Interactions**				
**RESIDUE-1**		**RESIDUE-2**		**Distance**
**Res Num**	**Res Name**	**Res Num**	**Res Name**	**(Å)**
520	ASP	24	HIS	5.77
550	ASP	41	ARG	6.16
**Potential Unfavorable Electrostatic Interactions**				
**RESIDUE-1**		**RESIDUE-2**		**Distance**
**Res Num**	**Res Name**	**Res Num**	**Res Name**	**(Å)**
493	LYS	24	HIS	9.32
**Potential Short Contacts**				
**RESIDUE-1**		**RESIDUE-2**		**Distance**
**Res Num**	**Res Name**	**Res Num**	**Res Name**	**(Å)**
436	ASP	58	ILE	2.22
440	LEU	57	SER	2.65
519	THR	24	HIS	2.22
546	GLN	23	ASN	2.6
547	LYS	63	ILE	2.43
554	ALA	42	LEU	2.94
554	ALA	42	LEU	2.88
554	ALA	42	LEU	1.70

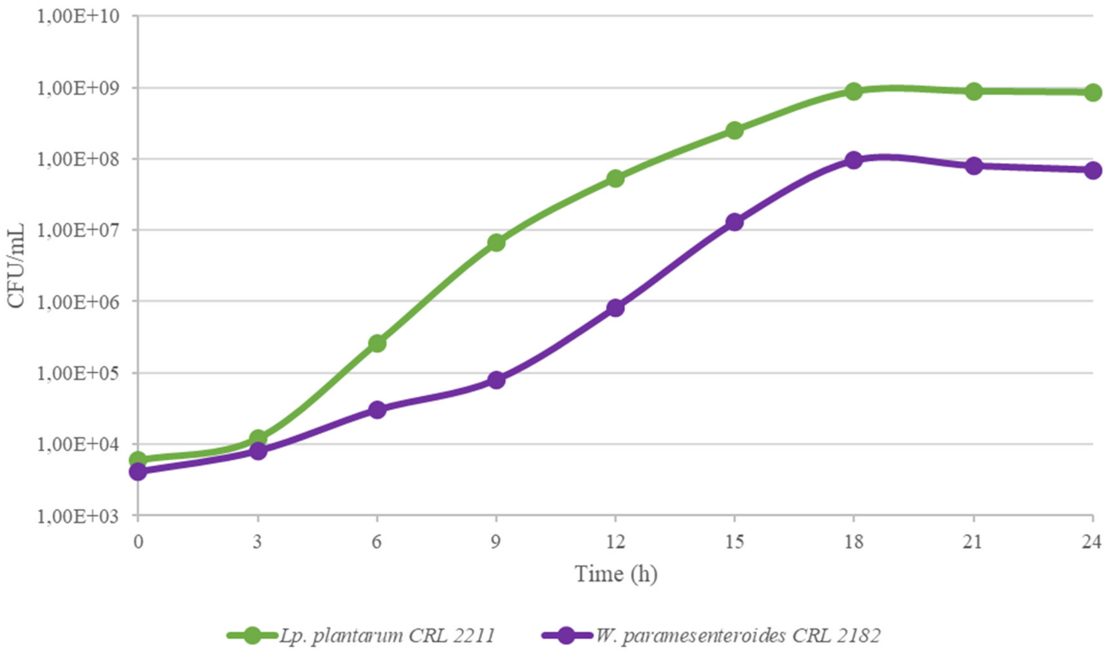

Figure S1. Growth curves (CFU/mL vs Time) in sterile bean extracts of LAB strains selected for flour fermentation: *Lp. plantarum* CRL 2211 and *W. paramesenteroides* CRL 2182.

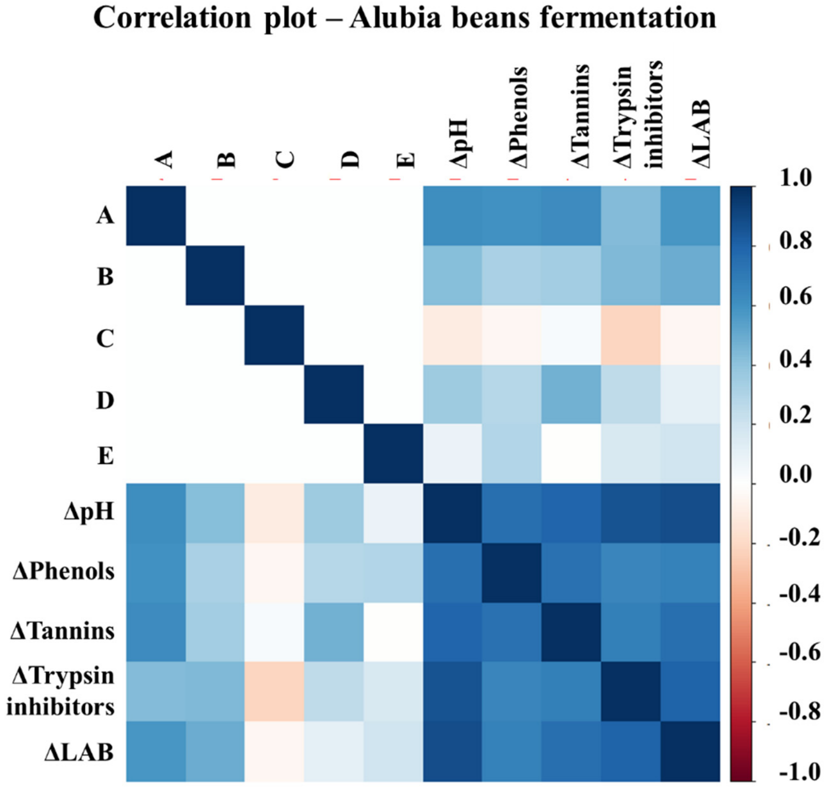

Figure S2. Correlations between experimental and response variables in Alubia beans fermentation process. Experimental variables selected were: A (*Lactiplantibacillus plantarum* CRL2211 addition), B (*Weissella paramesenteroides* CRL2182 addition), C (temperature, ºC), D (fermentation time, h), E (dough yield, %). Response variables studied were: vpH, ΔPhenols (total phenolic concentration, mg GAE/100g), ΔTannins (tannin concentration, mg GAE/100g), Δtrypsin inhibitors (concentration expressed as mg/g), ΔLAB (lactic acid bacteria count, Log CFU/g). Blue and red cells indicate positive and negative correlations expressed as Pearson correlation coefficients. Colour intensity is in proportion to magnitude. GAE: gallic acid equivalents

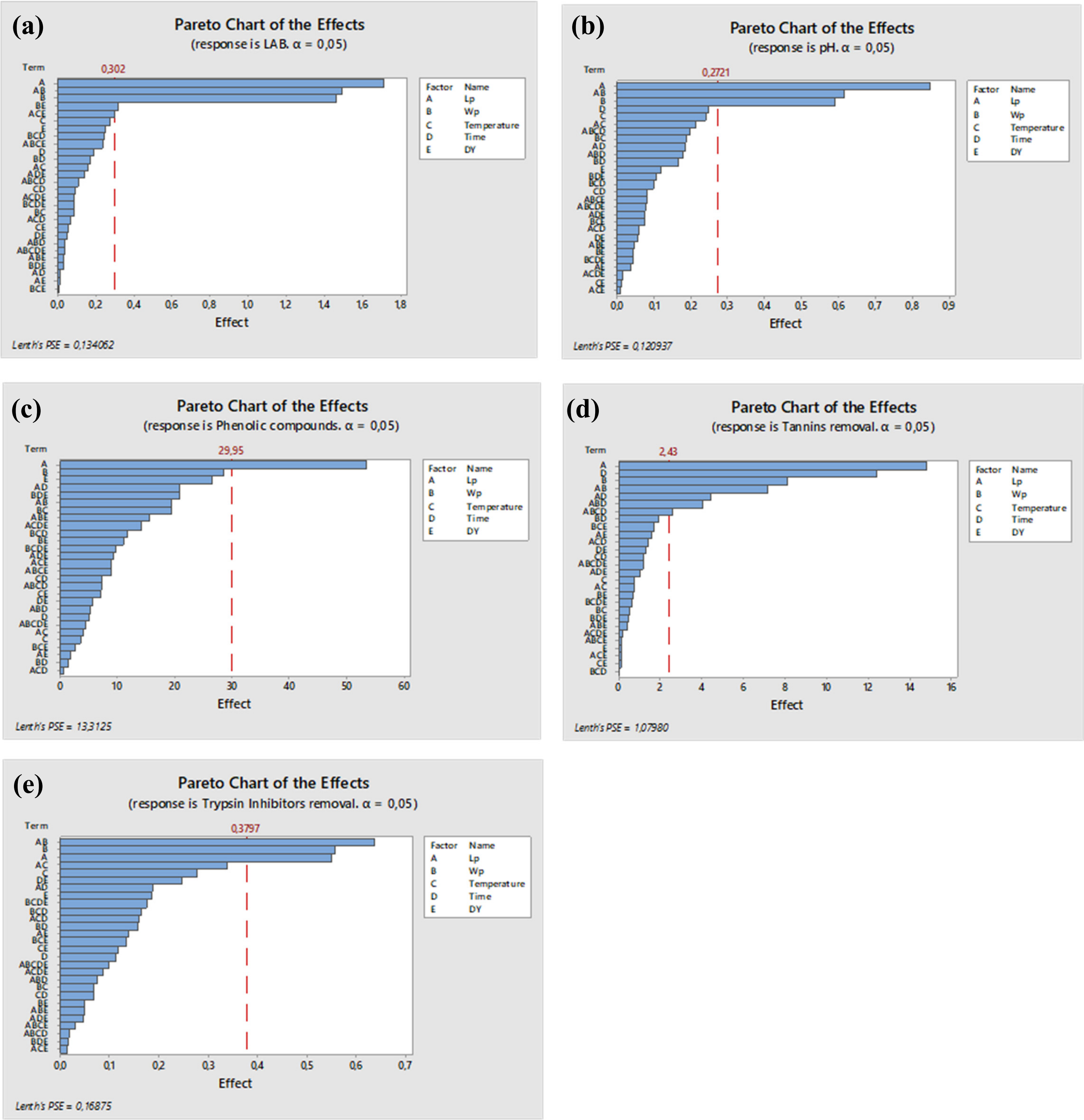

Figure S3. Pareto charts showing the effects of alubia beans fermentation conditions on the different responses: (a) Total LAB (lactic acid bacteria count, Log CFU/g), (b) Δ pH changes, (c) Δ TPC (total phenolic concentration, mg GAE/100g), (d) Δ tannin concentration (mg GAE/100g), **e)** Δ trypsin inhibitors (mg/g). Bars display the absolute value of the effects. Lines crossing the bars represents significant effects ($p < 0.05$) of process parameters. Any effect that extends beyond the reference line is potentially important

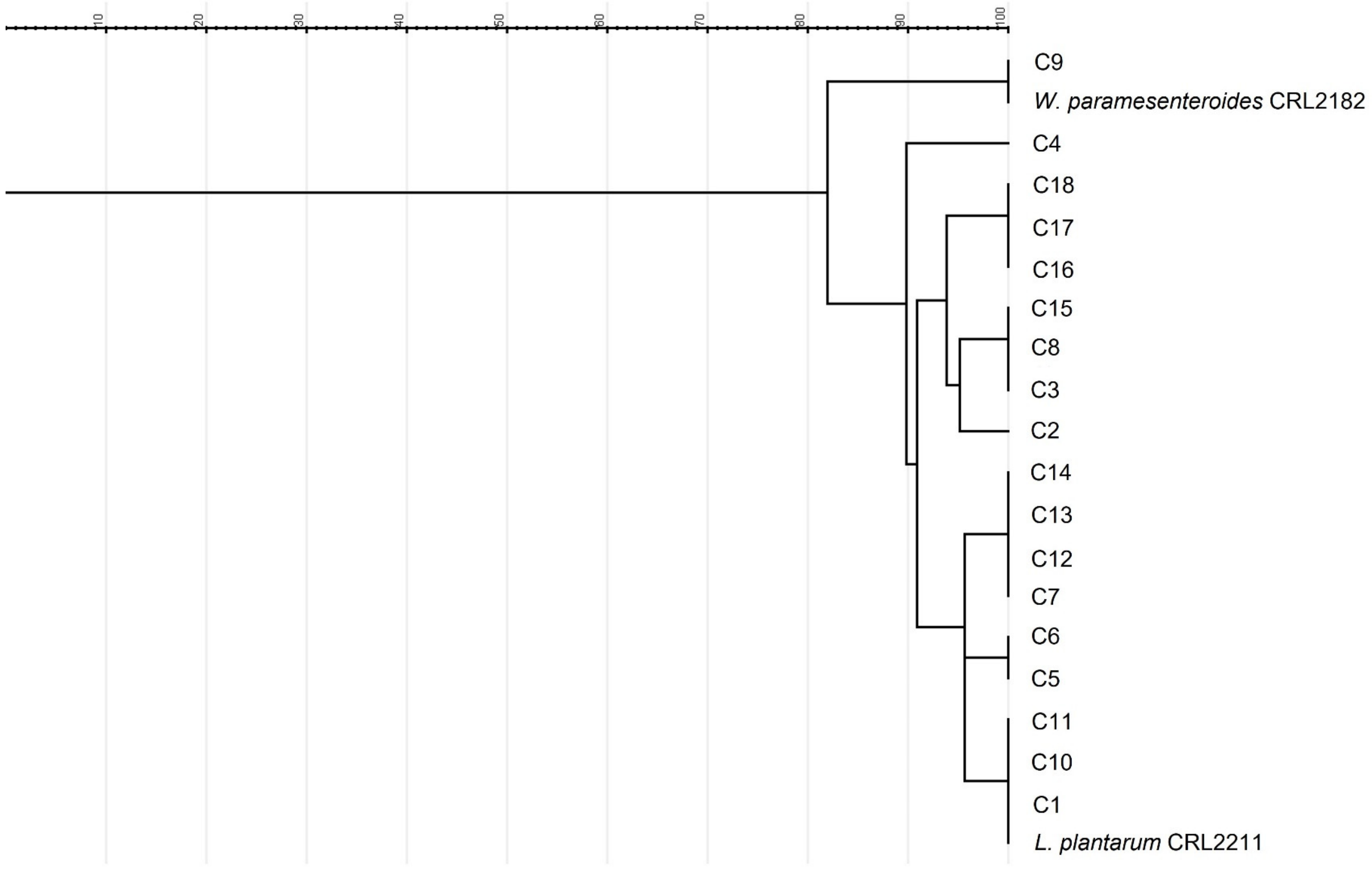

Figure S4. Dendrogram obtained by cluster analysis rep-PCR fingerprints of colonies isolated from fermented alubia beans flours with selected starter. The dendrogram is based on Pearson Coefficient of similarity with the unweighted pair group method with arithmetic averages clustering algorithm (UPGMA

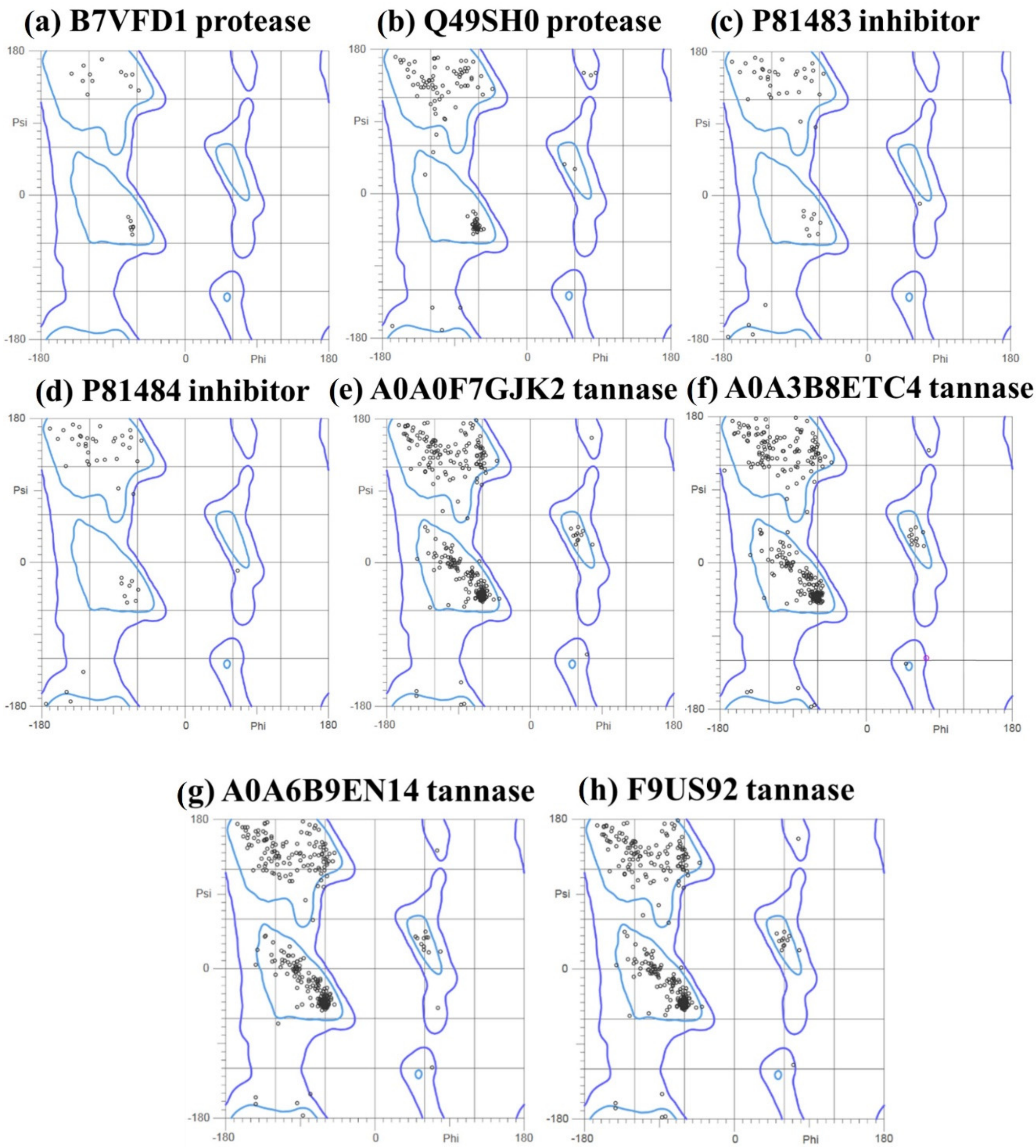

Figure S5. Ramachandran plots showing the values of phi (φ) and psi (ψ) angles of 3D structures from homology models of serine-type endopeptidase and tannases from *Lactiplantibacillus plantarum*, *Lactococcus lactis* and *Levilactobacillus brevis* and Bowman-Birk type proteinase inhibitors collected from UNIPROT (https://www.uniprot.org/ last accessed: 24/05/2024): (a) B7VFD1 protease, (b) Q49SH0 protease, (c) P81483 inhibitor, (d) P81484 inhibitor, (e) A0A0F7GJK2 tannase, (f) A0A3B8ETC4 tannase, (g) A0A6B9EN14 tannase, (h) F9US92 tannase. Each enzyme and inhibitor was assigned a database code from UNIPROT repository. These structures were used to simulate enzyme-substrate interactions in tannin hydrolysis and phenol release as well as the removal of trypsin inhibitors during alubia beans flour fermentation

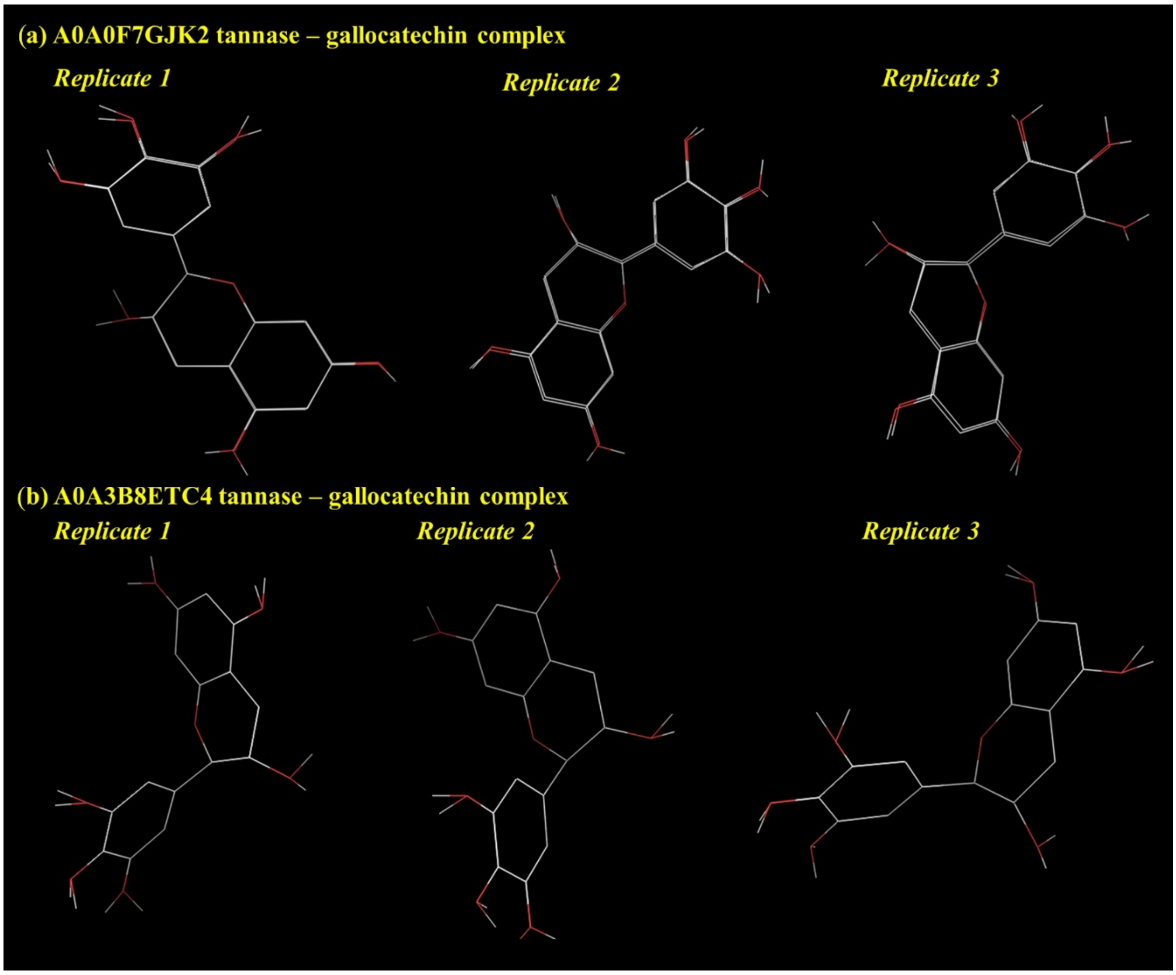

Figure S6. Redocking results of gallocatechin present in alubia beans flour and several tannases from *Lactiplantibacillus plantarum* and *Levilactobacillus brevis*: (a) A0A0F7GJK2, (b) A0A3B8ETC4, (c) A0A6B9EN14, (d) B3Y018, (e) F9US92. The similarity between the docked and bound conformations of each ligand in the crystal structure is illustrated

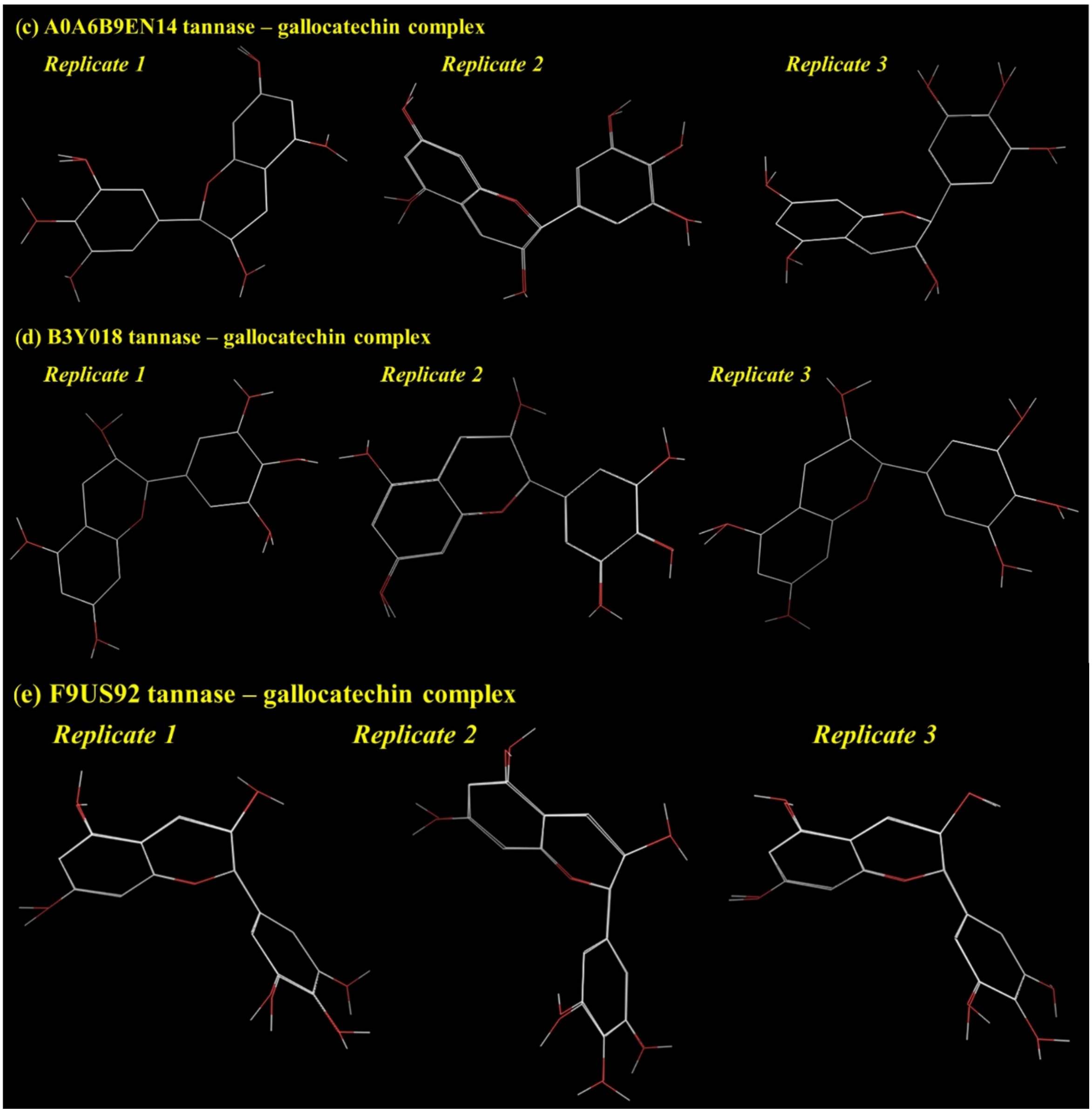

Figure S6. Cont.

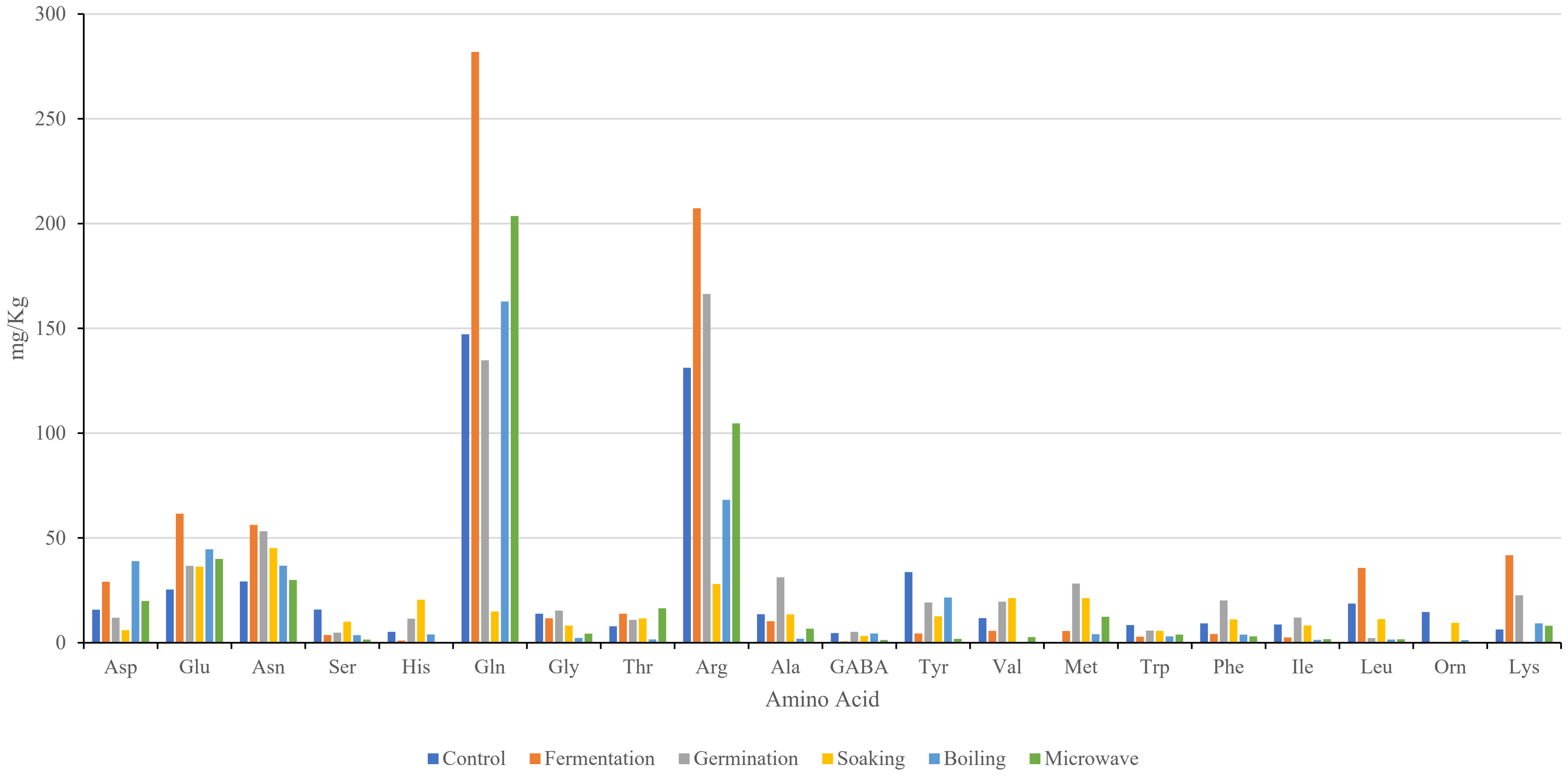

Figure S7. Concentration of free amino acids and derivates (mg/Kg) on alubia beans flours after different processing methods. Three letters for IUPAC code is used
